# Deciphering chemical diversity among five variants of *Abeliophyllum distichum* flowers through metabolomics analysis

**DOI:** 10.1002/pld3.616

**Published:** 2024-09-19

**Authors:** Yeong‐Geun Lee, Jeong Eun Kwon, Won‐Sil Choi, Nam‐In Baek, Se Chan Kang

**Affiliations:** ^1^ Graduate School of Biotechnology and Department of Oriental Medicine Biotechnology Kyung Hee University Yongin Korea; ^2^ National Instrumentation Center for Environmental Management Seoul National University Seoul Korea

**Keywords:** *Abeliophyllum distichum*, chemical mapping, metabolomics, miseon, morphological characteristic

## Abstract

*Abeliophyllum distichum* (Oleaceae), endemic to the Korean Peninsula and the sole member of its genus and species, possesses high scarcity value, escalating its importance under the Nagoya Protocol. Despite its significance, their metabolites and activities of *A. distichum* flowers remain unexplored. This study employs an integrated metabolomic approach utilizing NMR, LC/MS, GC/MS, and FTIR techniques to comprehensively analyze the metabolite profile of *A. distichum* flowers. By combining these methods, we identified 35 metabolites, 43 secondary metabolites, and 108 hydrophobic primary metabolites. Notably, distinct concentration patterns of these compounds were observed across five variants, classified based on morphological characteristics. Correlation analyses of primary and secondary metabolites unveiled varietal metabolic flux, providing insights into *A. distichum* flower metabolism. Additionally, the reconstruction of metabolic pathways based on dissimilarities in morphological traits elucidates variant‐specific metabolic signatures. These findings not only enhance our understanding of chemical differences between varieties but also underscore the importance of considering varietal differences in future research and conservation efforts.

## 1 INTRODUCTION

Protection of the sovereignty of biological resources in each country is important due to the recent Nagoya protocol (Kamau et al., 2010). The purpose of this protocol is the fair and equitable sharing of benefits arising from the use of genetic resources, which contributes to the conservation and sustainable use of biodiversity. Research using domestic native species became a key issue with this protocol in force.

At present, there are only six plants (*Abiliophyllum distichum* Nakai, *Echinosophora koreensis* Nakai, *Hanabusa asiatica* Nakai, *Pentactina rupicola* Nakai, *Megaleranthis saniculifolia* Ohwi, *Mankua chejuense* B.Y. Sun, M.H. Kim & C.H. Kim) out of 4159 homegrown plants in Korea that are monotaxons. Among the various domestic native plants, *A. distichum* Nakai (Oleaceae), known as white forsythia, is both one species and one genus and is grown only on the Korean Peninsula (Lee, 1983; Park et al., 2019). Due to its high scarcity and ecological and geographic value, the study of *A. distichum* has rarely been carried out compared with other plants. Previous phytochemical studies of these plants have focused on just the leaves, and they reported that four phenylethanoid glycosides and two flavonoids are components of these plants (Kuwajima et al., 1993; Li et al., 2013; Oh et al., 2003). In 2017, *A. distichum* was deleted from the list of endangered species by the Ministry of the Environment (Ministry of Environment, 2017) due to the development of a mass breeding technique (Lee et al., 2014, 2015).

**FIGURE 1:**
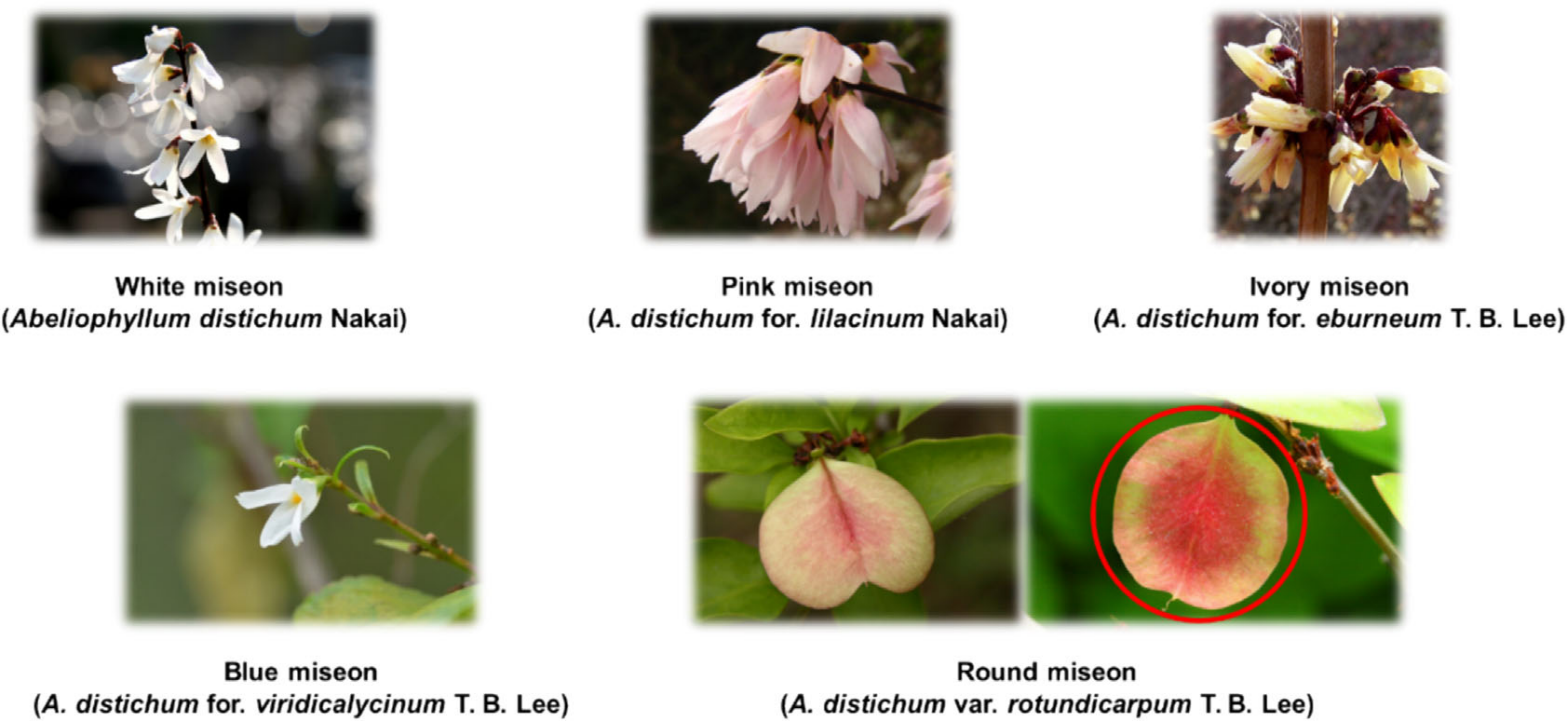
Appearances of *Abeliophyllum distichum* flowers.

At present, five variants of this plant have been reported: white miseon (*A. distichum* Nakai), pink miseon (*A. distichum* for. *lilacinum* Nakai), ivory miseon (*A. distichum* for. *eburneum* T. B. Lee), blue miseon (*A. distichum* for. *viridicalycinum* T. B. Lee), and round miseon (*A. distichum* var. *rotundicarpum* T. B. Lee) (Lee, 1976; Nakai, 1919, 1922). The variants were classified based only on morphological characteristics like the color of the petals and sepals or the shapes of the fruit (Figure 1). There are many opinions on the taxonomic identities of this plant, and some documents even suggest that each variant had the same taxa (Kim et al., 2002). Accordingly, phytochemical investigations and chemical maps for the variants should be valuable.

In this study, metabolomics was applied to estimate the dissimilarities in the chemical compositions of the variants of *A. distichum* flowers to understand the dissimilarities of the morphological characteristics. NMR spectroscopy, LC/MS, GC/MS, and FTIR were used as the main metabolomic tools.

Compared with other platforms, LC/MS is the most suitable platform for metabolomic studies of secondary metabolites (Bin et al., 2012; Rogachev & Aharoni, 2012). Accordingly, chemical mapping for classification using LC/MS on this plant will provide very useful data for understanding how classification in *A. distichum* could be expressed and associated with its morphological characteristics.

However, LC/MS‐based metabolomics is limited to secondary metabolites, and it was difficult to analyze the whole metabolome of five variants of *A. distichum*. In organisms, primary metabolites such as nucleotides, amino acids, organic acids, and sugars are directly related to the development, growth, and reproduction of cells, and so on (Kind et al., 2009). Thus, analysis of primary metabolites of this plant using another platform can help us to understand how metabolic flux is related to its morphological characteristics. There are many analytical methods for analyzing primary metabolites such as GC/MS, NMR, and CE/MS, along with others (Emwas et al., 2019). Among them, the GC/MS platform has many advantages, such as superior sensitivity, robustness, and reproducibility for small and hydrophilic molecules, including primary metabolites (Jonsson et al., 2006). Also, GC/MS‐based metabolomics that focuses on hydrophobic primary metabolites cannot measure all primary metabolites (especially the more polar ones).

NMR spectroscopy is one of the most powerful techniques for identifying absolute chemical structures using their chemical shifts, coupling constants, and coupling patterns (Smolinska et al., 2012). Moreover, intensities in NMR data are proportional to the molecular concentration, making quantification of all compounds possible even without calibration curves of each metabolite (Simmler et al., 2014). Specifically, NMR is able to reflect the real molecular concentrations of metabolites present in the whole organism. For this reason, NMR‐based metabolomics is used to determine the metabolites in numerous plant and animal sources in extracts and live organisms without purification and separation (Beger, 2013; Bharti et al., 2011; Cuperlovic‐Culf & Culf, 2016; Jia et al., 2015). Due to these advantages, metabolomic research using NMR has steadily increased over the past 15 years (Emwas et al., 2019). However, the application of NMR in the metabolomic study has some disadvantages, such as a lack of sensitivity and difficulty identifying overlapping peaks (Takis et al., 2019; Kim, Choi, & Verpoorte, 2010). However, there are some solutions to the shortcomings of NMR to a certain degree. A lack of sensitivity of NMR analysis can be improved with multiple scans and higher magnet field strengths. Overlapping peaks can be resolved using various 2D NMR techniques such as COSY, TOCSY, DOSY, NOESY, *J*‐resolved, HSQC, and HMBC (Emwas et al., 2019).

For this reason, there is no single tool used to analyze whole metabolites. In this study, we used a combination of NMR, LC/MS, and GC/MS for the complete identification of all metabolites, and quantification platforms were used for chemical mapping to determine the dissimilarities and chemical compositions of the metabolites (to reveal their metabolic flux) and reconstruct a metabolic pathway for understanding the dissimilarities in the morphological characteristics for the flower in five variants of *A. distichum*.

## 2 RESULTS AND DISCUSSION

### 2.1 Profiling of metabolites from the flowers in five variants of *A. distichum* using UHPLC‐TripleTOF‐ESI‐MSMS analysis

The metabolites of the flowers in five variants of *A. distichum* flowers were identified using UHPLC‐TripleTOF‐ESI‐MSMS analysis. Among the various extracted conditions of *A. distichum* flowers (50% to 100% MeOH), 80% aqueous MeOH was selected as the best extraction solvent in preliminary experiments (data not shown). UHPLC equipment and a 1.6‐μm particle size column was used for chromatographic analysis under a short analytical time for secondary metabolites. The solvent gradient elution was set as described in the Material and Method section to separate each metabolite effectively. Both ion modes (negative and positive) were tested for electrospray ionization (ESI) instruments (data not shown). Among them, all components of *A. distichum* flowers showed higher sensitivity in negative ion mode.

**FIGURE 2:**
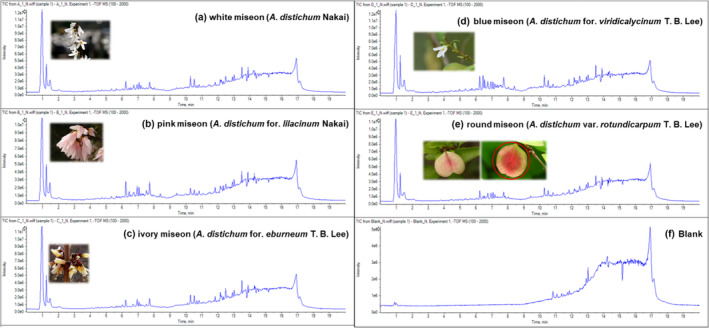
Representative total ion chromatograms (TIC) for five variants of *Abeliophyllum distichum* flowers. (a) White miseon ( *A. distichum* Nakai); (b) pink miseon ( *A. distichum* for. *lilacinum* Nakai); (c) ivory miseon ( *A. distichum* for. *eburneum* T. B. Lee); (d) blue miseon ( *A. distichum* for. *viridicalycinum* T. B. Lee); (e) round miseon ( *A. distichum* var. *rotundicarpum* T. B. Lee); (f) blank.

In this study, LC/MS spectra were recorded in quadruplicate. Figure 2 shows the representative total ion chromatograms (TIC) of different metabolites from five variants of *A. distichum* flowers. The intensities of peaks, especially those eluted from 6 to 8 min, were significantly different depending on each morphological characteristic. For instance, variants of colorless petals on white miseon and round miseon flowers showed similar aspects in each chromatogram. Chromatograms of blue miseon, which has blue sepals, showed different aspects compared with the others. Chromatograms of pink miseon showed strong intensities of each peak eluted from 6 to 8 min. These chromatograms indicated that each variant had different metabolic compositions based on their morphological characteristics, such as the color of the petals and sepals and the shape of the fruit.

Identification of each component was carried out based on a comparison of mass data for each peak with those in the NIST Library, HMDB, and MoNA export LC‐MS, MS‐MS Library; 250 components identified with scores higher than 0.8 were separated using UHPLC‐TripleTOF‐ESI‐MSMS in 15 min. Among them, 43 metabolites were selected according to the mass accuracy of each peak (values smaller than 7 ppm) as well as literature, isotopic pattern, and MSMS patterns (Table 1).

**TABLE 1:** The metabolites identified from the flowers in five variants of *Abeliophyllum distichum* using UHPLC‐TripleTOF‐ESI‐MSMS analysis.

No	RT(min)	Analyte name	Molecular formula	Molecular weight	Adduct	Theoretical m/z	Observed MS	Error(ppm)
1	0.918	5‐Acetylamino‐6‐formylamino‐3‐methyluracil	C_8_H_10_N_4_O_4_	226.0702	[M‐H]^−^	225.0629	225.0617	5.332
2	0.919	Glutamine	C_5_H_10_N_2_O_3_	146.0691	[M‐H]^−^	145.0618	145.0626	5.515
3	0.924	Mannose	C_6_H_12_O_6_	180.0634	[M‐H]^−^	179.0561	179.0564	1.675
4	0.924	Glucose	C_6_H_12_O_6_	180.0634	[M‐H]^−^	179.0561	179.0565	2.234
5	0.944	D‐gluconic acid	C_6_H_12_O_7_	196.0583	[M‐H]^−^	195.0510	195.0517	3.589
6	0.945	D‐xylonic acid	C_5_H_10_O_6_	166.0477	[M‐H]^−^	165.0405	165.0407	1.212
7	0.946	5‐*O*‐*p*‐coumaroylquinicacid	C_16_H_18_O_8_	338.1002	[M‐HCOO]^−^	383.0984	383.0994	2.610
8	0.949	4‐*O*‐caffeoylquinic acid	C_16_H_18_O_9_	354.0951	[M‐HCOO]^−^	399.0933	399.0952	4.761
9	0.962	Cellobiose	C_12_H_22_O_11_	342.1162	[M‐H]^−^	341.1089	341.1085	1.173
10	0.968	Quinic acid	C_7_H_12_O_6_	192.0634	[M‐H]^−^	191.0561	191.0567	3.140
11	0.993	Malic acid	C_4_H_6_O_5_	134.0215	[M‐H]^−^	133.0142	133.0149	5.263
12	1.006	2‐Chloro‐5‐nitro‐*N*‐phenyl‐benzamide	C_13_H_9_ClN_2_O_3_	276.0302	[M‐H]^−^	275.0229	275.0215	5.090
13	1.243	Citrate	C_6_H_8_O_7_	192.0270	[M‐H]^−^	191.0197	191.0196	0.524
14	1.245	Uridine	C_9_H_12_N_2_O_6_	244.0695	[M‐H]^−^	243.0623	243.0623	0.000
15	1.309	Adenosine	C_10_H_13_N_5_O_4_	267.0967	[M‐HCOO]^−^	312.0950	312.0947	0.961
16	1.365	Guanosine	C_10_H_13_N_5_O_5_	283.0917	[M‐H]^−^	282.0844	282.0842	0.709
17	2.005	2‐Methylglutaric acid	C_6_H_10_O_4_	146.0579	[M‐HCOO]^−^	191.0561	191.0564	1.570
18	3.795	Tryptophan	C_11_H_12_N_2_O_2_	204.0899	[M‐H]^−^	203.0826	203.0827	0.492
19	4.941	1‐*O*‐(*E*)‐caffeoyl‐*β*‐D‐glucopyranose	C_15_H_18_O_8_	326.1002	[M‐H]^−^	325.0929	325.0928	0.308
20	5.323	Reversine	C_21_H_27_N_7_O	393.2277	[M‐H]^−^	392.2204	392.2188	4.079
21	6.223	(−)‐Pinoresinol di‐*O*‐*β*‐D‐glucopyranose	C_32_H_42_O_16_	682.2473	[M‐H]^−^	681.2400	681.2420	2.936
22	6.438	1‐*O*‐(*E*)‐phenylethyl‐*β*‐D‐glucofuranosyl‐*O*‐(1 → 2)‐*β*‐D‐glucopyranoside	C_19_H_28_O_10_	416.1682	[M‐H]^−^	465.1610	415.1603	1.505
23	6.530	1‐*O*‐(2*E*)‐6‐hydroxy‐2,6‐dimethyl‐2,7‐octadienoyl‐*β*‐D‐glucopyranoside	C_16_H_26_O_8_	346.1628	[M‐HCOO]^−^	391.1610	391.1612	0.511
24	6.664	Calendoside III	C_27_H_30_O_16_	610.1534	[M‐H]^−^	609.1461	609.1474	2.134
25	6.713	Echinacoside	C_35_H_46_O_20_	786.2852	[M‐H]^−^	785.2510	785.2539	3.693
26	6.739	Amarantholidol A glycoside	C_21_H_38_O_9_	434.2516	[M‐HCOO]^−^	479.2498	479.2500	0.417
27	6.906	Forsythoside F	C_34_H_44_O_19_	756.2477	[M‐H]^−^	755.2404	755.2429	3.310
28	6.929	(−)‐8‐Hydroxypinoresinol 4‐*O*‐*β*‐D‐glucopyranose	C_26_H_32_O_12_	536.1894	[M‐HCOO]^−^	581.1876	581.1882	1.032
29	6.941	Rutin	C_27_H_30_O_16_	61.0153	[M‐H]^−^	609.1461	609.1476	2.462
30	7.269	Acteoside	C_29_H_36_O_15_	624.2054	[M‐H]^−^	623.1981	623.1988	1.123
31	7.516	Nicotiflorin	C_27_H_30_O_15_	594.1584	[M‐H]^−^	593.1512	593.1521	1.517
32	7.720	Pinoresinol‐4‐*O*‐glucoside	C_26_H_32_O_11_	520.1945	[M‐H]^−^	519.1872	519.1883	2.119
33	8.204	3‐*O*‐*β*‐D‐glucopyranosylcucurbic acid	C_18_H_30_O_8_	374.1941	[M‐HCOO]^−^	419.1923	419.1924	0.239
34	10.106	Naringenin	C_15_H_12_O_5_	272.0685	[M‐H]^−^	271.0612	271.0615	1.107
35	10.282	(10*E*,15*Z*)‐9,12,13‐trihydroxyoctadeca‐10,15‐dienoic acid	C_18_H_32_O_5_	328.2250	[M‐H]^−^	327.2177	327.2184	2.139
36	12.155	Cafestol	C_20_H_28_O_3_	316.2039	[M‐HCOO]^−^	361.2020	361.1996	6.644
37	12.339	(2*S*)‐1‐*O*‐linolenoyl‐3‐*O*‐*β*‐D‐galactopyranosyl‐sn‐glycerol	C_27_H_46_O_9_	514.3142	[M‐HCOO]^−^	559.3124	559.3136	2.145
38	12.491	Coronaric acid	C_18_H_32_O_3_	296.2351	[M‐H]^−^	295.2279	295.2287	2.710
39	12.537	Corosolic acid	C_30_H_48_O_4_	472.3553	[M‐H]^−^	471.3480	471.3477	0.636
40	12.624	*N*‐cyclopentyl‐1‐[5,6‐dimethyl‐1‐(1‐methylethyl)‐1H‐benzimidazol‐2‐yl]‐4‐piperidinecarboxamide	C_23_H_34_N_4_O	382.2733	[M‐H]^−^	381.2660	381.2642	4.721
41	12.634	13‐HOTrE(r)	C_18_H_30_O_3_	294.2915	[M‐H]^−^	293.2122	293.2122	0.000
42	13.516	2‐Hydroxypalmitic acid	C_16_H_32_O_3_	272.2351	[M‐H]^−^	271.2279	271.2285	2.212
43	13.822	Ursolic acid	C_30_H_48_O_3_	456.3604	[M‐H]^−^	455.3537	455.3532	1.098

*Note*: Each compound was identified by comparison of database, NIST Library, HMDB, and MoNA export LC‐MS, MS‐MS Library.

### 2.2 Profiling of primary metabolites from the flowers in five variants of *A. distichum* using GC‐MS analysis

**FIGURE 3:**
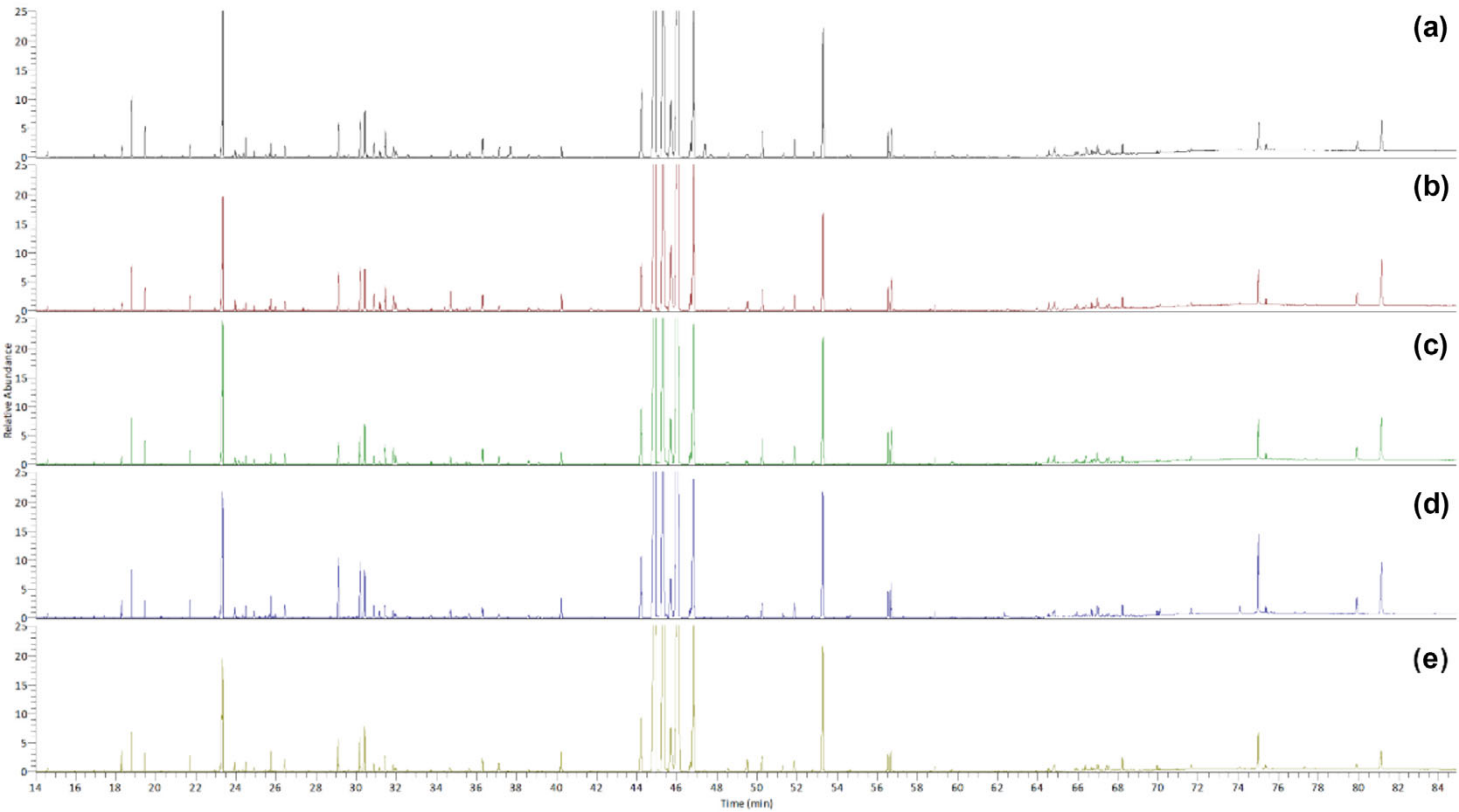
Representative total ion chromatograms (TIC) for five variants of *Abeliophyllum distichum* flowers. (a) White miseon ( *A. distichum* Nakai); (b) pink miseon ( *A. distichum* for. *lilacinum* Nakai); (c) ivory miseon ( *A. distichum* for. *eburneum* T. B. Lee); (d) blue miseon ( *A. distichum* for. *viridicalycinum* T. B. Lee); (e) round miseon ( *A. distichum* var. *rotundicarpum* T. B. Lee).

The identification of hydrophobic primary metabolites was run in triplicates based on the TIC from the measurement of five variants of *A. distichum* flowers using GC coupled with triple quadrupole‐mass spectrometry (QqQ/MS). Figure 3 shows the representative TIC of diverse primary metabolites emitted from five variants of *A. distichum* flowers. The intensities of the peaks were significantly different depending on the sample, indicating that each variant had different metabolic compositions as well as different morphological characteristics. Each peak was identified by matching their spectra with a NIST library and published literature as well as analyzing the retention indices calculated against *n*‐alkanes (C_7_–C_30_) (i.e., retention time and relative retention time). They were confirmed through analysis of fragmentation patterns in mass spectra. Sugar derivatives, such as hexose‐type sugars, pentose‐type sugars, and sugar alcohol, were gauged by the retention index (RI) values in the literature (Choi et al., 2013; Medeiros & Simoneit, 2007; Wagner et al., 2003; Xia et al., 2018).

A total of 108 primary metabolites were identified and quantified in GC/MS analysis of five variants of *A. distichum* flowers, including seven organic alcohols and their aldehydes, 11 organic acids, 20 amino acids, four amino alcohols, five nucleic acids, 11 fatty acids, 31 sugars, including its alcohols and acids, and five others. RT, relative RT (RRT), RI, quantification ion (QI), and mass fragments of analytes are illustrated in Table 2.

**TABLE 2:** The primary metabolites identified from the flowers in five variants of *Abeliophyllum distichum* using GC‐MS analysis.

No	Compound		RT^a^	RRT^b^	RI^c^	QI^d^	Mass fragment [*m/z* (relative intensity by comparison of biggest peak)]^e^
1	Ethylene glycol	2TMS	14.63	0.277	<900	**147**	147 (100), 73 (89), 75 (52), 77 (29), 103 (21), 128 (18), 148 (17), 66 (16), 45 (16), 191 (15)
2	Propylene glycol	2TMS	15.10	0.286	<900	**117**	73 (100), 117 (88), 77 (67), 75 (46), 147 (45), 79 (18), 44 (17), 66 (17), 47 (13), 45 (13)
3	Cyclohexanol	1TMS	15.47	0.293	1006	**75**	75 (100), 73 (48), 77 (34), 157 (34), 129 (30), 44 (13), 79 (13), 45 (11)
4	Carbamate	3TMS	15.96	0.302	1022	**147**	73 (100), 147 (51), 77 (41), 75 (30), 44 (18), 79 (17), 174 (15), 100 (14), 72 (14), 45 (12)
5	Ethanolamine‐1	2TMS	16.11	0.305	1026	**147**	102 (100), 73 (66), 147 (21)
6	1‐Cyclohexene‐1‐ol	1TMS	16.94	0.321	1053	**75**	75 (100), 73 (82), 155 (38), 127 (32), 170 (29), 169 (21), 45 (20), 77 (15), 147 (13), 117 (12)
7	Acetic acid	2TMS	17.46	0.331	1069	**73**	73 (100), 147 (76), 66 (19), 75 (19), 45 (14), 177 (14), 148 (13)
8	L‐valine	1TMS	17.92	0.34	1084	**72**	72 (100), 73 (21), 75 (21), 55 (18)
9	L‐alanine	2TMS	18.32	0.347	1096	**116**	116 (100), 73 (60), 147 (15), 117 (11)
10	1‐*N*‐carboxy glycine	3TMS	19.53	0.370	1136	**73**	73 (100), 147 (63), 133 (25), 59 (15), 45 (15), 100 (15), 72 (15), 86 (13), 75 (12), 220 (11)
11	*β*‐Lactate	2TMS	19.57	0.371	1137	**177**	147 (100), 73 (58), 75 (35), 131 (19), 177 (16), 148 (16), 219 (15), 44 (14), 45 (14), 98 (12)
12	L‐leucine	1TMS	20.00	0.379	1151	**86**	86 (100), 75 (38), 44 (32), 73 (22), 45 (11)
13	L‐proline	1TMS	20.63	0.391	1172	**70**	70 (100), 75 (20), 73 (11)
14	L‐isoleucine	1TMS	20.65	0.391	1173	**86**	86 (100), 73 (33), 75 (31), 44 (17), 69 (14), 70 (11)
15	Malonic acid	2TMS	21.34	0.404	1195	**147**	147 (100), 73 (66), 75 (28), 148 (15), 66 (14), 45 (13)
16	L‐valine	2TMS	21.78	0.413	1210	**144**	144 (100), 73 (73), 218 (19), 147 (14), 145 (13)
17	2‐Phenylethanol	1TMS	22.19	0.42	1225	**103**	73 (100), 103 (65), 179 (60), 75 (49), 77 (18), 105 (18), 44 (17), 79 (16), 91 (15), 45 (15)
18	4‐Hydroxybutyric acid	2TMS	22.30	0.423	1229	**147**	147 (100), 73 (65), 75 (52), 117 (20), 148 (17), 44 (17), 45 (15), 233 (15), 77 (11)
19	Urea	2TMS	22.40	0.424	1232	**189**	147 (100), 73 (71), 75 (61), 44 (53), 189 (53), 171 (28), 77 (27), 79 (26), 74 (21), 45 (21)
20	L‐serine	2TMS	22.94	0.435	1251	**116**	73 (100), 116 (66), 132 (52), 75 (35), 57 (19), 45 (16), 89 (15), 74 (14), 103 (13), 147 (11)
21	Ethanolamine‐2	3TMS	23.23	0.440	1261	**174**	174 (100), 73 (56), 86 (21), 100 (19), 175 (17), 147 (15)
22	Phosphoric acid	3TMS	23.30	0.441	1263	**299**	299 (100), 73 (87), 300 (24), 314 (16), 133 (15), 301 (14), 45 (13)
23	Glycerol	3TMS	23.34	0.442	1265	**147**	73 (100), 147 (51), 205 (34), 103 (27), 117 (24), 158 (19), 133 (14), 218 (12)
24	1,2,3‐Butantriol	3TMS	23.83	0.451	1282	**117**	73 (100), 117 (90), 116 (37), 147 (35), 205 (24), 75 (21), 103 (17), 133 (13), 118 (12), 45 (12)
25	L‐isoleucine	2TMS	23.96	0.454	1286	**158**	158 (100), 73 (78), 218 (18), 159 (14), 147 (12)
26	Nicotic acid	1TMS	24.08	0.456	1291	**180**	180 (100), 136 (59), 106 (42), 78 (42), 51 (14), 181 (13), 73 (13), 75 (12)
27	L‐proline‐1	2TMS	24.14	0.457	1293	**142**	142 (100), 73 (55), 143 (11)
28	L‐glycine	3TMS	24.35	0.461	1300	**174**	174 (100), 73 (79), 147 (27), 86 (26), 175 (19), 248 (18), 100 (14), 45 (12), 59 (12)
29	Succinic acid	2TMS	24.50	0.464	1306	**147**	147 (100), 73 (73), 75 (28), 148 (15)
30	Glyceric acid	3TMS	24.91	0.472	1321	**73**	73 (100), 147 (43), 189 (28), 103 (16), 292 (14), 133 (13), 102 (13), 75 (11), 45 (11)
31	Uracil	2TMS	25.19	0.477	1331	**99**	99 (100), 241 (86), 73 (74), 147 (59), 255 (44), 256 (37), 113 (32), 45 (29), 242 (20)
32	Fumaric acid	2TMS	25.48	0.483	1342	**245**	245 (100), 73 (88), 147 (53), 75 (44), 246 (21), 45 (19), 143 (18), 83 (13)
33	L‐serine	3TMS	25.73	0.487	1351	**204**	73 (100), 204 (87), 218 (54), 100 (23), 147 (17), 205 (16), 75 (12), 45 (11), 219 (10)
34	Nonanoic acid	1TMS	25.82	0.489	1354	**215**	75 (100), 73 (90), 215 (54), 117 (52), 129 (19), 132 (15), 55 (14), 131 (14), 74 (12), 45 (11)
35	Erythoronic acid	2TMS	26.20	0.496	1369	**147**	73 (100), 147 (83), 75 (24), 101 (22), 45 (19), 103 (16), 148 (15), 247 (14), 116 (13), 87 (13)
36	L‐theronine	3TMS	26.44	0.501	1377	**218**	73 (100), 218 (34), 219 (31), 117 (27), 147 (20), 101 (18), 75 (14), 57 (13), 74 (10)
37	*β*‐Alanine	3TMS	27.63	0.523	1422	**174**	73 (100), 174 (65), 248 (49), 147 (40), 86 (19), 75 (18), 45 (16), 59 (15), 100 (15), 249 (14)
38	L‐asparatic acid	3TMS	28.72	0.544	1463	**232**	73 (100), 232 (59), 147 (55), 70 (26), 100 (16), 45 (11)
39	Malic acid	3TMS	29.10	0.551	1477	**73**	73 (100), 147 (40), 233 (13), 75 (11)
40	Butanal	3TMS	29.41	0.557	1488	**103**	73 (100), 217 (39), 103 (28), 147 (27), 117 (17), 205 (13), 204 (11)
41	L‐threitol	4TMS	29.65	0.562	1497	**217**	73 (100), 147 (42), 217 (36), 103 (28), 205 (22), 117 (17), 204 (13)
42	L‐asparatic acid	3TMS	30.04	0.569	1510	**232**	73 (100), 232 (67), 100 (26), 147 (16), 218 (14), 75 (13), 233 (12), 74 (11)
43	L‐proline‐2	2TMS	30.18	0.572	1514	**156**	156 (100), 73 (79), 147 (18), 157 (13), 45 (11)
44	*γ*‐Aminobutyric acid (GABA)	3TMS	30.43	0.577	1522	**174**	174 (100), 73 (71), 147 (31), 86 (18), 175 (18), 304 (17), 75 (12)
45	1‐Deoxypentitol	4TMS	30.57	0.579	1527	**217**	73 (100), 117 (59), 103 (29), 147 (25), 217 (22), 219 (13), 75 (12), 129 (11)
46	L‐threonic acid‐1	4TMS	30.68	0.581	1530	**292**	73 (100), 147 (34), 75 (19), 292 (16), 220 (12), 44 (11), 117 (11)
47	L‐threonic acid‐2	4TMS	31.18	0.591	1546	**292**	73 (100), 147 (36), 292 (17), 220 (13), 205 (12), 117 (11)
48	L‐tartaric acid‐3	4TMS	32.56	0.617	1589	**117**	73 (100), 117 (28), 44 (27), 75 (23), 147 (14), 292 (14), 219 (13), 129 (12), 74 (11), 45 (11)
49	Glutaric acid	3TMS	32.67	0.619	1592	**247**	73 (100), 147 (44), 75 (33), 244 (25), 247 (20), 44 (18), 45 (15), 115 (14), 116 (14), 231 (11)
50	L‐glutamic acid	3TMS	33.31	0.631	1609	**246**	73 (100), 246 (78), 75 (32), 128 (29), 147 (25), 247 (16), 84 (16), 156 (16), 44 (14), 45 (14)
51	L‐phenylalanine	2TMS	33.76	0.640	1620	**218**	73 (100), 218 (66), 192 (44), 147 (23), 75 (15), 193 (15), 267 (13), 45 (13), 100 (12), 219 (12)
52	Lyxose^f^	4TMS	34.40	0.652	1635	**217**	73 (100), 103 (66), 217 (35), 147 (25), 307 (18)
53	Arabinose‐1^f^	4TMS	34.72	0.658	1643	**103**	73 (100), 103 (74), 217 (36), 147 (25), 307 (22)
54	Ribose^f^	4TMS	35.02	0.664	1650	**103**	73 (100), 103 (71), 217 (35), 147 (28), 307 (20), 75 (11)
55	L‐asparagine	3TMS	35.34	0.670	1658	**116**	73 (100), 116 (44), 75 (29), 231 (23), 132 (19), 147 (17), 188 (14), 74 (14), 204 (13), 45 (12)
56	Rhamnose^f^	4TMS	37.60	0.712	1710	**117**	73 (100), 117 (65), 75 (27), 147 (18), 44 (16)
57	Ribitol^f^	5TMS	37.68	0.714	1712	**103**	73 (100), 217 (37), 103 (33), 147 (27), 205 (18), 129 (12)
58	Fucose^f^	4TMS	37.99	0.72	1718	**117**	73 (100), 117 (99), 147 (27), 75 (18), 160 (15), 103 (14), 217 (14), 44 (11), 133 (10)
59	Ribonic acid	5TMS	39.27	0.744	1746	**103**	73 (100), 103 (32), 147 (25), 217 (18), 292 (16), 333 (14), 75 (14)
60	L‐glutamine	3TMS	40.23	0.762	1767	**156**	73 (100), 156 (82), 155 (33), 75 (29), 245 (18), 147 (18), 74 (14), 157 (12), 45 (11)
61	L‐tartaric acid‐2	4TMS	41.78	0.792	1800	**117**	73 (100), 117 (57), 147 (31), 292 (19), 333 (18), 75 (11)
62	Quinic acid	5TMS	44.22	0.838	1854	**147**	73 (100), 147 (31), 345 (29), 204 (21), 255 (16), 191 (13)
63	Fructose^g^	5TMS	44.90	0.851	1869	**103**	73 (100), 103 (79), 217 (33), 147 (21), 307 (14)
64	Glucose^h^	5TMS	45.31	0.858	1878	**103**	73 (100), 103 (23), 147 (21), 217 (17), 205 (12), 306 (12)
65	Mannose‐1^i^	5TMS	45.70	0.866	1887	**160**	73 (100), 205 (44), 319 (38), 147 (35), 217 (23), 160 (19), 103 (16), 320 (11)
66	Galactose‐1^i^	5TMS	46.10	0.873	1896	**319**	73 (100), 319 (60), 205 (53), 147 (40), 160 (30), 217 (20), 320 (19), 103 (15), 206 (11), 117 (10)
67	Sorbose^f^	5TMS	46.67	0.884	1909	**205**	73 (100), 205 (51), 147 (39), 319 (34), 103 (29), 217 (24), 160 (18), 117 (10)
68	Mannose‐2^i^	5TMS	46.83	0.887	1913	**205**	73 (100), 205 (37), 319 (37), 147 (30), 103 (21), 160 (21), 217 (13), 320 (11)
69	Galactinol^f^	6TMS	47.41	0.898	1927	**205**	73 (100), 147 (28), 205 (27), 103 (22), 319 (22), 217 (21)
70	Mannitol^f^	6TMS	47.70	0.904	1934	**204**	73 (100), 147 (28), 205 (22), 204 (20), 217 (20), 103 (19), 319 (16), 75 (12)
71	Myo‐inositol‐1^f^	6TMS	48.49	0.919	1953	**318**	73 (100), 147 (30), 217 (29), 318 (23), 305 (22), 191 (18)
72	Glucuronic acid^g^	6TMS	50.25	0.952	1996	**333**	73 (100), 147 (38), 292 (21), 333 (20), 205 (17), 217 (15), 103 (14), 319 (11)
73	1‐Methyl hexose	5TMS	51.29	0.972	2026	**220**	204 (100), 73 (88), 147 (24), 205 (24), 220 (20), 217 (19), 319 (13)
74	Palmitic acid	1TMS	51.86	0.983	2044	**117**	117 (100), 73 (90), 75 (65), 313 (49), 132 (48), 129 (43), 145 (30), 43 (25), 55 (20), 41 (17)
75	Myo‐inositol‐2^f^	6TMS	53.26	1.009	2086	**217**	73 (100), 217 (62), 147 (50), 305 (47), 191 (33), 318 (27), 204 (15), 306 (14), 265 (13), 218 (13)
76	Margaric acid	1TMS	54.04	1.024	2112	**117**	73 (100), 75 (76), 117 (63), 129 (28), 327 (25), 132 (24), 55 (20), 145 (20), 57 (19), 43 (17)
77	Arabinose‐2^h^	5TMS	54.50	1.033	2129	**319**	73 (100), 147 (23), 319 (20), 75 (19), 205 (18), 44 (16), 103 (15), 217 (11)
78	Stearic acid‐1	1TMS	56.44	1.069	2202	**341**	73 (100), 75 (68), 117 (56), 129 (29), 132 (25), 43 (25), 341 (21), 55 (19), 44 (18), 145 (16)
79	Linoleic acid	1TMS	56.54	1.071	2206	**81**	73 (100), 75 (97), 67 (65), 81 (64), 95 (39), 55 (38), 79 (37), 82 (29), 129 (28), 117 (25)
80	Linolenic acid	1TMS	56.69	1.074	2213	**79**	75 (100), 73 (96), 79 (90), 67 (51), 95 (49), 93 (46), 81 (39), 80 (35), 55 (34), 91 (32)
81	Oleic acid	1TMS	56.85	1.077	2219	**117**	73 (100), 75 (77), 117 (37), 55 (32), 129 (30), 69 (21), 81 (20), 41 (18), 96 (18), 67 (17)
82	Stearic acid‐2	1TMS	57.31	1.086	2239	**117**	73 (100), 75 (76), 117 (70), 132 (31), 129 (30), 341 (28), 43 (24), 55 (22), 145 (19), 41 (15)
83	Glyceryl glycoside‐1	6TMS	58.55	1.109	2293	**147**	73 (100), 204 (82), 103 (25), 75 (20), 147 (20), 205 (17), 217 (13), 44 (13)
84	Glyceryl glycoside‐2	6TMS	58.87	1.115	2307	**204**	73 (100), 204 (97), 147 (25), 217 (23), 103 (23), 205 (20), 129 (13), 75 (12)
85	Glyceryl glycoside‐3	6TMS	59.70	1.131	2348	**204**	73 (100), 204 (67), 147 (24), 75 (20), 103 (18), 205 (16), 217 (15), 44 (11), 129 (11), 156 (11)
86	Glyceryl glycoside‐4	6TMS	59.77	1.132	2351	**204**	73 (100), 204 (80), 147 (21), 103 (19), 205 (18), 75 (17), 217 (16)
87	Uronic acid‐1	5TMS	60.10	1.139	2368	**217**	73 (100), 217 (30), 147 (24), 204 (22), 75 (21), 103 (18), 44 (11)
88	Diethylhexyl adipate		60.47	1.146	2386	**129**	129 (100), 57 (42), 70 (34), 55 (33), 71 (30), 112 (29), 147 (26), 43 (24), 41 (23), 111 (20)
89	5‐Methyluridine	3TMS	60.58	1.148	2391	**217**	73 (100), 217 (49), 147 (26), 75 (24), 207 (21), 103 (19), 129 (16), 44 (14), 133 (12), 218 (11)
90	Monolinolein	2TMS	61.42	1.164	2436	**132**	73 (100), 207 (38), 75 (35), 117 (33), 204 (26), 129 (24), 147 (21), 44 (20), 132 (17), 43 (16)
91	Uridine		61.51	1.165	2441	**218**	73 (100), 217 (49), 147 (17), 103 (17), 75 (16), 218 (11)
92	Galactose‐2^i^	5TMS	62.32	1.181	2484	**105**	204 (100), 73 (56), 105 (49), 223 (20), 217 (20), 205 (19), 147 (17), 103 (10)
93	Monopalmitin	2TMS	63.92	1.211	2577	**371**	73 (100), 371 (49), 147 (39), 57 (27), 75 (25), 43 (23), 71 (20), 129 (19), 55 (19), 103 (17)
94	Sucrose^i^	8TMS	64.54	1.223	2614	**204**	73 (100), 204 (74), 217 (29), 117 (20), 363 (20), 147 (19), 205 (15), 273 (13)
95	Adenosine	4TMS	64.75	1.227	2628	**236**	73 (100), 230 (41), 236 (35), 217 (24), 147 (18), 245 (17), 207 (16), 103 (14), 192 (13), 74 (10)
96	Lactulose^f^	8TMS	65.30	1.237	2662	**363**	73 (100), 204 (48), 207 (36), 147 (29), 217 (20), 117 (13), 205 (12), 75 (12), 103 (12), 363 (11)
97	Sucrose^f^	8TMS	66.24	1.255	2722	**217**	73 (100), 204 (53), 147 (36), 207 (36), 217 (28), 361 (20), 103 (18), 205 (17), 75 (12), 191 (12)
98	Lactose^f^	8TMS	66.38	1.258	2731	**361**	73 (100), 204 (48), 147 (34), 361 (34), 217 (32), 205 (20), 103 (18), 207 (15), 129 (13), 191 (10)
99	Acubin	6TMS	66.47	1.259	2737	**361**	73 (100), 361 (35), 191 (25), 147 (22), 217 (20), 103 (16), 204 (14), 75 (11), 362 (11), 129 (11)
100	Maltose^f^	8TMS	66.66	1.263	2750	**131**	204 (100), 73 (74), 147 (21), 205 (19), 207 (17), 217 (17), 131 (14), 103 (13), 107 (12), 75 (11)
101	Turanose^i^	8TMS	66.79	1.265	2759	**361**	73 (100), 204 (36), 207 (30), 147 (30), 217 (21), 103 (18), 361 (16), 205 (13), 191 (11)
102	Monostearin	2TMS	66.94	1.268	2769	**399**	73 (100), 399 (40), 147 (35), 57 (28), 43 (24), 129 (22), 71 (21), 75 (20), 55 (18), 103 (17)
103	Cellobiose^f^	8TMS	67.02	1.270	2774	**107**	204 (100), 73 (87), 147 (28), 217 (23), 205 (21), 207 (19), 107 (16), 131 (14), 103 (14), 75 (12)
104	Palatinose^i^	8TMS	67.43	1.278	2801	**103**	73 (100), 204 (62), 217 (52), 103 (49), 147 (37)
105	Mycose^f^	8TMS	67.56	1.280	2811	**361**	73 (100), 204 (66), 207 (30), 147 (30), 361 (23), 217 (23), 205 (15), 103 (12), 191 (11), 129 (9)
106	Isomaltose^i^	8TMS	68.20	1.292	2856	**361**	73 (100), 204 (60), 147 (34), 217 (33), 361 (23), 207 (22), 103 (16), 129 (16), 205 (16), 160 (12)
107	Glycopyranosyl cyclohexanehexol	9TMS	69.93	1.325	2939	**217**	73 (100), 204 (79), 217 (29), 147 (25), 207 (22), 191 (22), 205 (17), 103 (13), 129 (12)
108	Gentiobiose^f^	8TMS	74.98	1.421	>3000	**204**	204 (100), 73 (40), 205 (20), 147 (13), 217 (12)
ID^j^	Fluoranthene		52.78	1.000	2072	**202**	202 (100), 200 (19), 203 (17), 101 (15), 201 (14), 100 (13)

^a^ Retention time (min).

^b^ Relative retention time, retention time of analyte/retention time of fluoranthene.

^c^ Retention indices calculated against *n*‐alkanes (C_7_–C_30_).

^d^ Quantification ion (*m/z*), specific mass ion used for quantification.

^e^ Lists of the representative ion peaks, which have intensities relatively greater than 10% of the largest peak.

^f^ Refer to the RI values in Medeiros and Simoneit (2007).

^g^ Refer to the RI values in Wagner et al. (2003).

^h^ Refer to the RI values in Xia et al. (2018).

^i^ Refer to the RI values in Choi et al. (2013).

^j^ Internal standard.

### 2.3 Profiling of primary metabolites from five variants of the flowers in five variants of *A. distichum* using NMR analysis

The primary metabolites in five variants of *A. distichum* flowers were identified using NMR analysis. Six kinds of deuterated solvents (D_2_O, CD_3_OD, DMSO‐*d*_6_, acetone‐*d*_6_, pyridine‐*d*_5_, and CDCl_3_) are commonly used in NMR experiments. These solvents have different polarities, and solvents are chosen based on the solubility of the target component. Preliminary experiments were performed using these deuterated solvents to identify the most suitable extract conditions to provide samples high in primary metabolites. Among the various extracts using several deuterated NMR solvents of *A. distichum* flowers (Figure S1), D_2_O and CD_3_OD, which are highly polar solvents, effectively extracted primary metabolites such as organic acids, sugar, and amino acids in the preliminary experiments.

**FIGURE 4:**
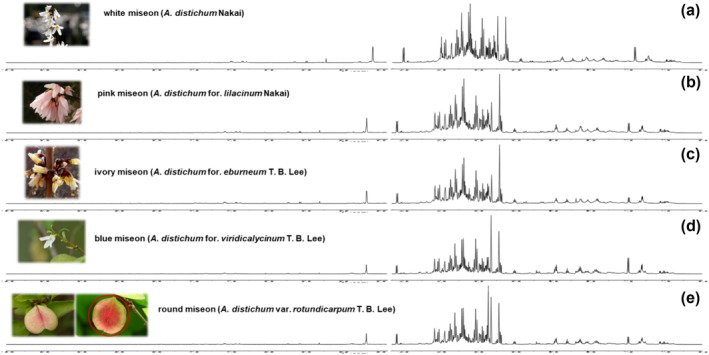
Representative ^1^H‐NMR spectra of flowers of white miseon (*Abeliophyllum distichum*) analyzed with 1:1 mixture of CD_3_OD and D_2_O. (a) White miseon ( *A. distichum* Nakai); (b) pink miseon ( *A. distichum* for. *lilacinum* Nakai); (c) ivory miseon ( *A. distichum* for. *eburneum* T. B. Lee); (d) blue miseon ( *A. distichum* for. *viridicalycinum* T. B. Lee); (e) round miseon ( *A. distichum* var. *rotundicarpum* T. B. Lee).

**FIGURE 5:**
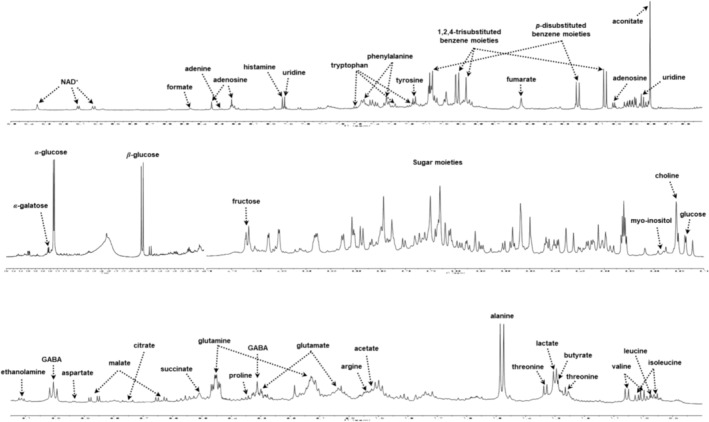
Representative ^1^H‐NMR spectrum of flowers of white miseon (*Abeliophyllum distichum*) analyzed with 1:1 mixture of CD_3_OD and D_2_O.

Thus, samples were extracted and analyzed with deuterated 50% aqueous methanol to obtain comprehensive NMR spectra of the metabolites of *A. distichum* flowers. Figure 4 shows the representative ^1^H‐NMR spectra of each variant, which showed significant differences depending on each morphological characteristic. Further confirmation of each metabolite in the extract was achieved using 2D spectra (including COSY [Figure S2], HSQC [Figure S3], HMBC [Figure S4], TOCSY [Figure S5], and *J*‐reserved [Figure S6]) and a comparison with their literature values (Abbas et al., 2018; Dai et al., 2010; Grauso et al., 2019; Kim, Choi, & Verpoorte, 2010; Kumar et al., 2019; Li et al., 2018; ). We identified 35 metabolites, including amino acids, nucleic acids, organic acids, secondary metabolites, and sugars. These are shown in the three main regions of a representative ^1^H‐NMR spectrum of *A. distichum* flowers in Figure 5 and Table 3.

**TABLE 3:** The metabolites identified from the flowers in five variants of *Abeliophyllum distichum* using NMR analysis.

Classification	Compound	Chemical shift and multiplicity (ppm, coupling pattern, *J* in Hz)
Nucleic acids	NAD^+^	9.35 (s), 9.10 (d, 6.0), 9.02 (d, 6.0)
	Adenosine	8.34 (s), 8.22 (s)
	Adenine	8.28 (d, 9.0), 6.01 (d, 6.0)
	Histamine	7.93 (s)
	Uridine	7.92 (s), 5.84 (d, 7.8)
Amino acids	Tryptophan	7.50 (d, 7.8), 7.27 (m), 7.17 (d, 7.8)
	Phenylalanine	7.46 (m), 7.33 (m)
	Tyrosine	7.16 (d, 8.4)
	Asparate	2.93 (d, 3.6)
	Glutamine	2.45 (dt, 7.8, 3.0), 2.10 (m)
	Proline	2.34 (d, 4.8)
	Arginine	1.94 (m)
	Alanine	1.48 (d, 7.2)
	Threonine	1.32 (d, 7.2), 1.26 (d, 7.2)
	Valine	1.05 (d, 7.2), 1.00 (d, 7.2)
	Isoleucine	1.02 (d, 7.2), 0.95 (d, 7.2)
	Leucine	0.98 (d, 7.2), 0.96 (d, 7.2)
Sugars	*α*‐Galatose	5.21 (d, 3.6)
	*α*‐Glucose	5.19 (d, 3.6), 3.81, 3.70, 3.47, 3.37, 3.19 (dd, 8.4, 7.8)
	*β*‐Glucose	4.60 (d, 7.8), 3.87, 3.70, 3.42, 3.37, 3.19 (dd, 8.4, 7.8)
	Fructose	4.07 (d, 3.0), 3.78, 3.64
Organic acids	Formate	8.41 (s)
	Fumarate	6.55 (s)
	Aconitate	5.80 (s)
	Malate	2.86 (dd, 10.8, 1.8), 2.63 (dd, 10.8, 5.4)
	Citrate	2.75 (d, 12.0)
	Succinate	2.50 (s)
	Glutarate	2.30 (m), 2.03 (m)
	Acetate	1.91 (s)
	Lactate	1.30 (d, 7.2)
	Butyrate	1.29 (d, 7.2)
Amino alcohols	Ethanolamine	3.11 (t, 5.4)
	GABA	3.00 (t, 7.2), 2.31
	Choline	3.21 (s)
Alcohol	Myo‐inositol	3.23 (d, 7.8)

### 2.4 Multivariate data analyses of metabolites using LC/MS

**FIGURE 6:**
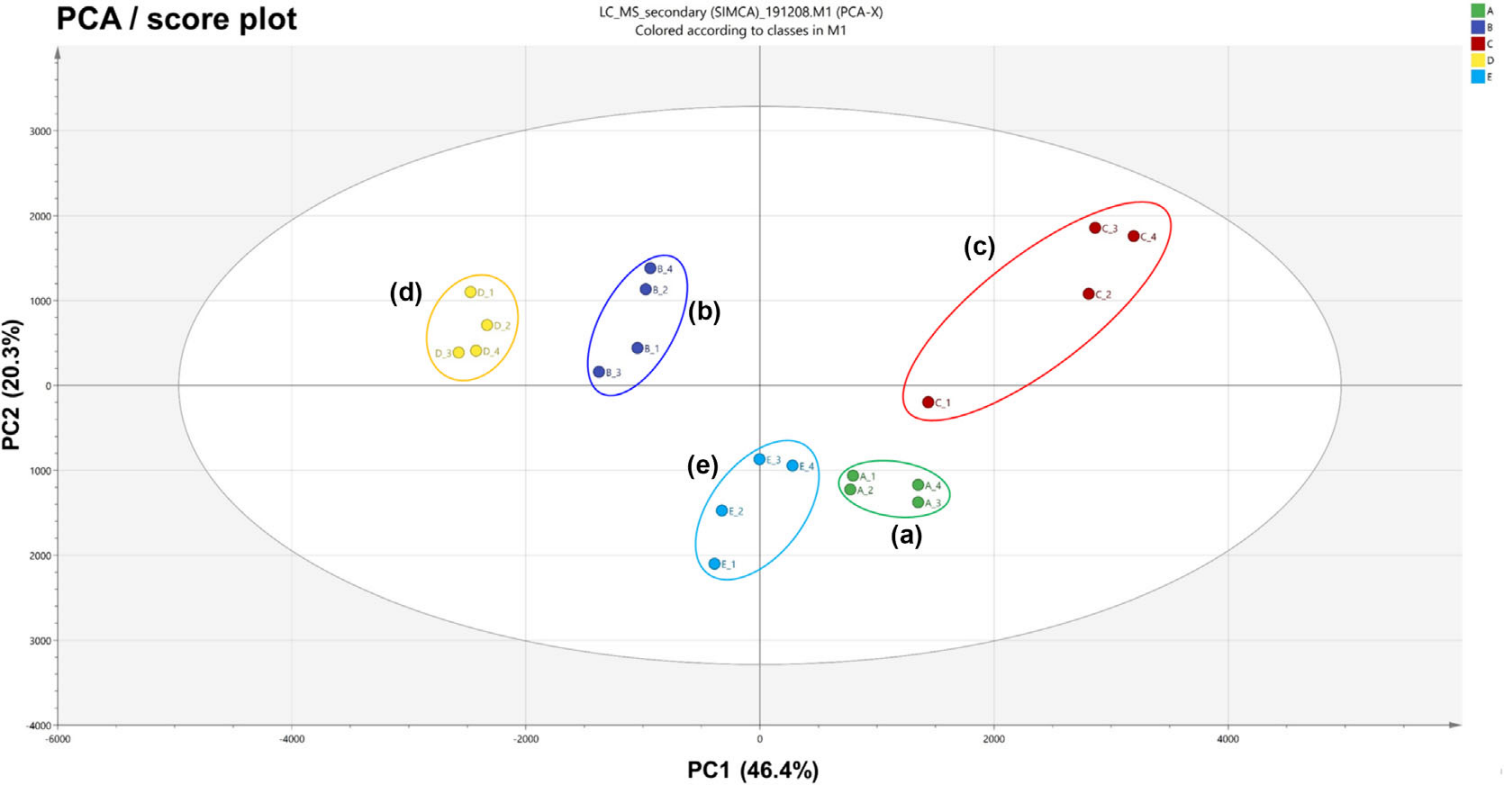
PCA score plot obtained from LC‐MS data on five variants of *Abeliophyllum distichum* flowers. (a) White miseon, (b) pink miseon, (c) ivory miseon, (d) blue miseon, (e) round miseon.

To understand how the classification of the flowers in five variants of *A. distichum* could be expressed and correlated to morphological characteristics, multivariate data analyses were performed on the metabolites identified in Materials and Methods. The metabolite fingerprinting of five variants of *A. distichum* flowers (Figure 6) was carried out with PCA, an unsupervised multivariate pattern recording method, to visualize the dissimilarities based on the chromatographic pattern efficiently. Mean‐centered and par‐scaled (scaled to the square root of SD) mathematical methods were performed to pretreat the data sets resulting from the above samples using the SIMCA‐P 14.1 software.

The PCA and PLS‐DA results show a distinct separation between all five variants of *A. distichum* flowers. The PCA score plots (Figure 6) representing an analysis derived from the negative ionization mode describe 66.7% of the total variance in which optimal segregation was achieved between the principal component 1 (PC1, 46.4%) and the principal component 2 (PC2, 20.3%), where PC1 was the key component for sample separation. Pink miseon and blue miseon were clearly separated from the others by PC1. Also, white miseon and round miseon, which have white petals and purple sepals, were clearly separated from the others by the PC2. The loading plots (Figure S7) and biplot (Figure S8) of the PCA results were consistent with their respective score plots, in which significant intensities of each metabolite variable for their correlative clusters reflected variations in their metabolite fingerprints. Based on PC1 in loading plots, pink miseon, which has unique petal colors, and blue miseon, which has a blue color on the sepals, showed higher content of lignans ((−)‐pinoresinol di‐*O*‐*β*‐D‐glucopyranose, (−)‐8‐hydroxypinoresinol 4‐*O*‐*β*‐D‐glucopyranose, and pinoresinol 4‐*O*‐glucoside), phenylethanoid glycosides (echinacoside, forsythoside F, and acteoside), and phenolic glucosides (1‐*O*‐(*E*)‐caffeoyl‐*β*‐D‐glucopyranose, and 1‐*O*‐(*E*)‐phenylethyl‐*β*‐D‐glucofuranosyl‐*O*‐(1 → 2)‐*β*‐D‐glucopyranoside) than others. On the other hand, flavonoids (calendoside III, rutin, nicotiflorin, and naringenin) were more prevalent in pink miseon and blue miseon compared with the others. The PC2 loading plot shows that all secondary metabolites were higher in pink and ivory miseon, which have colored petals, and blue miseon, which have different colors on the sepal, as compared with white miseon and round miseon, which have white petals and purple sepals. The biplot (Figure S8) of the PCA results also showed the distribution of markers in variants of *A. distichum* flowers. The preferential distribution of flavonoids in the first quadrant of the biplot primarily accounted for the differences in ivory miseon. Also, phenylethanoid glycosides, lignans, and phenolic glucosides distributed in the second quadrant of biplots account for the variations in pink and blue miseon. Otherwise, no secondary metabolite was distributed in the third and fourth quadrants of the biplots, accounting for the variations in white and round miseon. This alignment indicates that variations in secondary metabolite fingerprints have an effect on the morphological characteristics of *A. distichum* flowers such as the shape of the fruit and the colors of the sepals and petals.

Although the score plots of PCA/PLS‐DA results provide some idea of grouping, the available principal components are limited because only three of them can be graphically presented. Also, the score plots do not provide any information on the similarities between groups (Kim, Choi, & Verpoorte, 2010; Kim, Saifullah, et al., 2010). Therefore, we constructed a dendrogram for hierarchical cluster analysis (HCA) to reveal the similarities between the five variants of *A. distichum* flowers using 43 metabolites obtained from LC/MS results. As shown in Figure S9, white miseon and round miseon, which have white petals and similar shapes, showed higher similarity than the other variants. Ivory miseon, which has ivory petals, was clearly different from the other variants. These results indicate that various secondary metabolites (i.e., phenylethanoid glycosides, flavonoids, phenyl ethanoid, and phenyl propanoids) had a major influence on chemical mapping and helped us to understand the morphological characteristics.

### 2.5 Multivariate data analyses of metabolites using GC/MS

**FIGURE 7:**
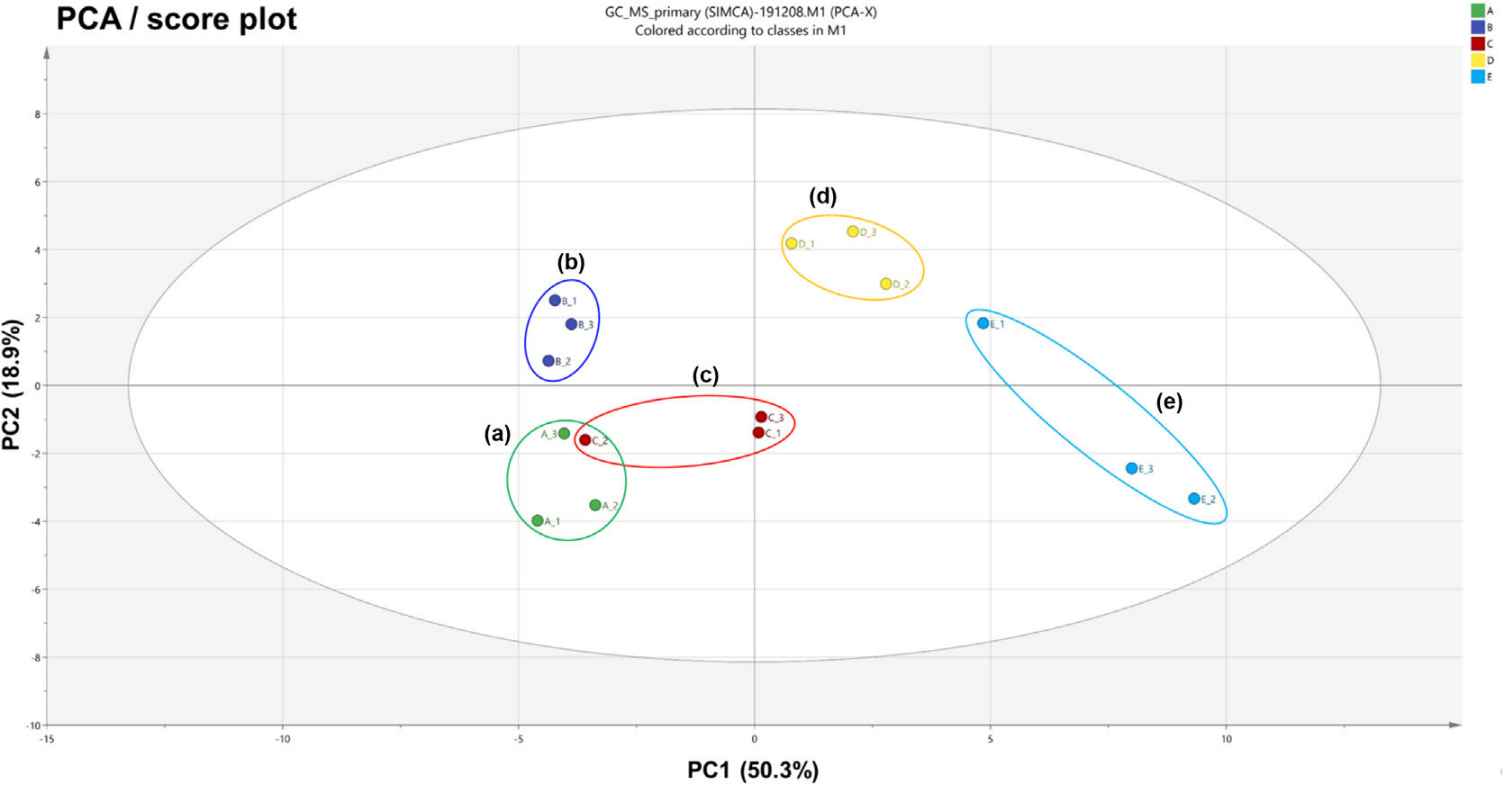
PCA score plot obtained from GC/MS results on five variants of *Abeliophyllum distichum* flowers. (a) White miseon, (b) pink miseon, (c) ivory miseon, (d) blue miseon, (e) round miseon.

Before the multivariate data analyses, each peak identified as a primary metabolite (Table 2) was normalized to fluoranthene as an internal standard. The PCA and PLS‐DA results show distinct separations between all five variants of *A. distichum* flowers, indicating that each primary metabolite was related to the phenotype of each variant. The PCA result derived from components of each variant is shown in the score plot (Figure 7) together with the loading plot (Figure S10), constituting the first principal component (PC 1, 50.3%) and second principal component (PC2, 18.9%). These described 69.2% of the total variance in the optimal separation of data. Variants based on different colors of sepals or fruit shapes (blue miseon and round miseon) and others were clearly segmented by the PC1 and by the PC2, where pink and blue miseon were clearly separated from the others. The loading plots (Figure S10) of PCA results were consistent with their respective score plots, which described the dissimilarities of hydrophobic primary metabolite characteristics based on their correlative clusters, highlighting variations in their fingerprints. Based on the PC1 in the loading plot, blue miseon and round miseon, which have different fruit shapes or sepal colors showed higher contents of seven amino acids (L‐isoleucine, L‐valine, L‐serine, L‐glutamine, L‐proline, L‐alanine, and GABA), four hexose types of sugars (glucose, mannose, fructose, and gentiobiose), two sugar derivatives (myo‐inositol and quinic acid), and one organic acid (malate) than others. On the other hand, one amino acid (L‐serine), one organic acid (malonic acid), seven 6‐deoxyhexose or pentose types of sugars (arabinose, ribose, rhamnose, lyxose, fucose, and sucrose), three sugar alcohols (galactinol, ribitol, and mannitol), and four fatty acids (stearic acid, palmitic acid, and linoleic acid) were high in white, pink, and ivory miseon compared with other variants. On the basis of PC2 in the loading plot, glycerol, two organic acids (succinic acid and phosphoric acid), four sugars (mannose, fructose, glucose, and glucuronic acid), three sugar alcohols (ribitol, galactinol, and myo‐inositol), and two fatty acids (palmitic acid and stearic acid) were high in white, ivory, and round miseon compared with others that have different colors of petals (pink miseon) or sepals (blue miseon). Otherwise, one amino acid (L‐proline), one organic acid (malate), and two sugars (gentiobiose and arabinose) were highly concentrated in blue and pink miseon. PLS‐DA analysis was performed to further elucidate the segregation of primary metabolites from each variant, especially white and ivory miseon (Xia et al., 2017). PLS‐DA results (Figures S11 and S12) show a total variance of 68.6% and showed many of the same aspects as the PCA results. This alignment indicates that primary metabolite fingerprints are deeply related to the morphological characteristics of *A. distichum* flowers such as the fruit shape and color of sepals and petals.

### 2.6 Multivariate data analyses of metabolites using NMR

**FIGURE 8:**
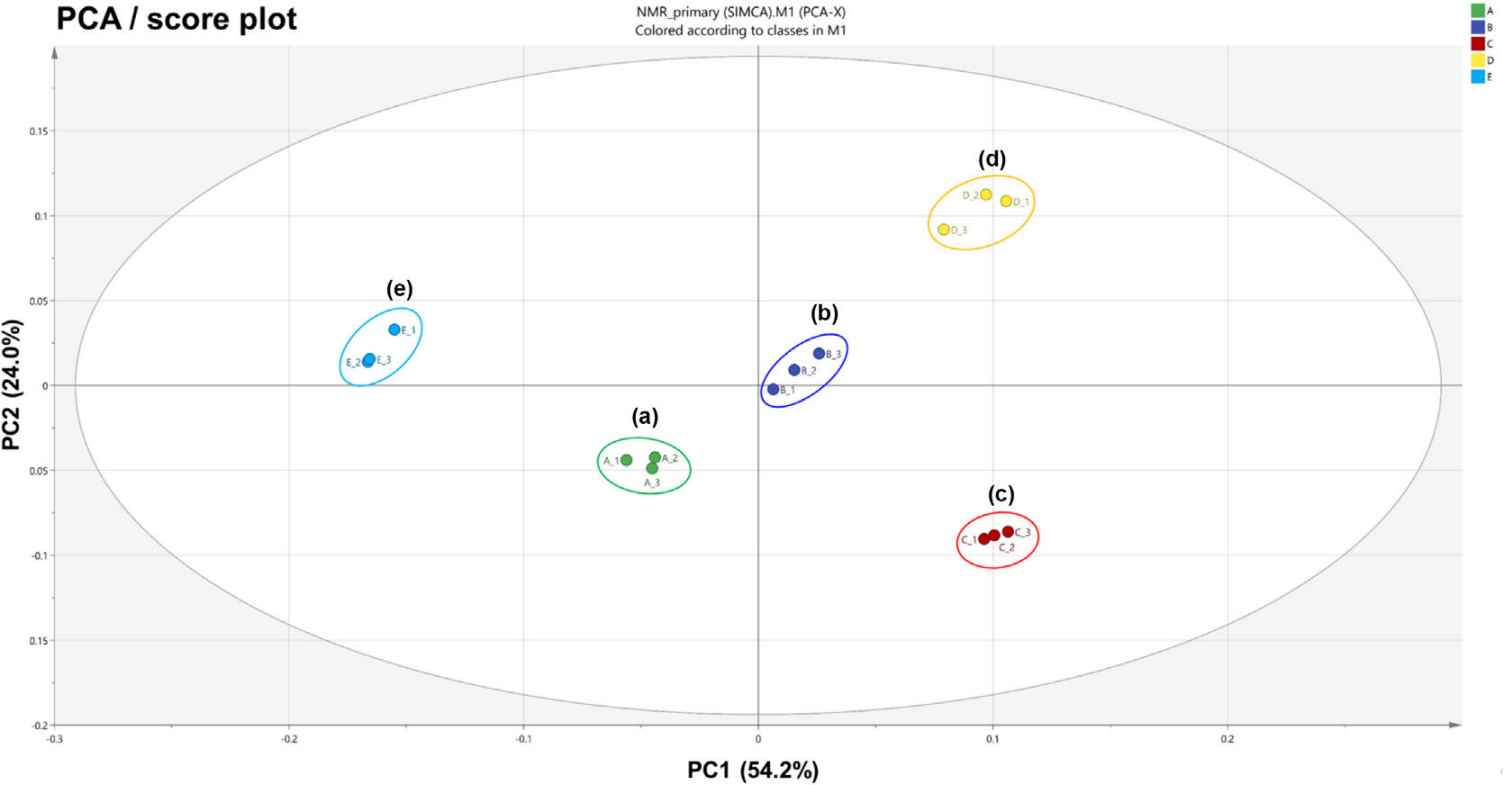
PCA score plot obtained from NMR result on five variants of *Abeliophyllum distichum* flowers. (a) White miseon, (b) pink miseon, (c) ivory miseon, (d) blue miseon, (e) round miseon.

The metabolite fingerprints of five variants of *A. distichum* flowers (white miseon, pink miseon, ivory miseon, blue miseon, and round miseon) (Figure 8) were obtained with PCA, a method of unsupervised multivariate projection, to effectively describe the dissimilarities based on the NMR data patterns (Jonsson et al., 1991). Mathematical methods of scaling and mean‐centering the data set to unit variance resulting from the above samples were performed by the SIMCA‐P 14.1 software. Before multivariate data analyses, each peak of the identified primary metabolites (Figure 5) was normalized to TSP as an internal standard. The PCA and PLS‐DA results show distinct separations between all five variants of *A. distichum* flowers, indicating that metabolites were related to the phenotype of each variant. The PCA result derived from the components of each variant shows the score plot (Figure 8) together with the loading plot (Figure S13), constituting the first principal component (PC 1, 54.2%) and second principal component (PC2, 24.0%). These described 78.2% of the total variance in which the data were optimally separated. White miseon and round miseon, which have the same colors of petals and sepals, and others were clearly distinguished by the PC1 and by the PC2. Blue miseon and round miseon were also clearly separated from the others.

The loading plots (Figure S13) of PCA results were consistent with their respective score plots, in which the described dissimilarities of primary metabolic characteristics from their correlative clusters were observed by variations in their fingerprints. Based on PC1 in the loading plot, white miseon and round miseon showed higher contents of sugars (*α*‐glucose, *β*‐glucose, and *α*‐galactose) and amino acid (alanine) than others. On the other hand, more amino acids (glutamine, proline, arginine, threonine, and leucine) and amino alcohols (choline) were contained in pink, ivory, and blue miseon compared with white and round miseon. On the basis of PC2 in the loading plot, three amino acids (glutamine, alanine, and valine) were high in blue miseon and round miseon as compared with the others. Otherwise, three amino acids (tryptophan, tyrosine, and glutarate), one nucleic acid (uridine), and glucose (both of *α*‐ and *β*‐configuration) were highly concentrated in pink and round miseon. This alignment indicates that the fingerprints of metabolites are deeply related to the morphological characteristics of *A. distichum* flowers.

## 3 EXPERIMENTAL PROCUDERS

### 3.1 Plant materials and sample preparation

The flowers of *Abeliophyllum distichum* were provided by the Miseonnamu‐maeul Agricultural Association Corporation (CEO: Jongtae Woo), Goesan‐gun, Chungcheongbuk‐do, Republic of Korea in April 2017. Five variants, white miseon (*A. distichum* Nakai, KHU‐NPCL‐201704‐01), pink miseon (*A. distichum* for. *lilacinum* Nakai, KHU‐NPCL‐201704‐02), ivory miseon (*A. distichum* for. *eburneum* T. B. Lee, KHU‐NPCL‐201704‐03), blue miseon (*A. distichum* for. *viridicalycinum* T. B. Lee, KHU‐NPCL‐201704‐04), and round miseon (*A. distichum* var. *rotundicarpum* T. B. Lee, KHU‐NPCL‐201704‐05), were identified by Prof. Dae‐Keun Kim, College of Pharmacy, Woosuk University, Jeonju, South Korea. Voucher specimens (KHU‐NPCL‐201704‐01‐05) have been deposited at the Natural Products Chemistry Laboratory, Kyung Hee University.

Five dried variants of *A. distichum* flowers (5 g) were ground in a freezer mill (6875D, SPEX SamplePrep, Metuchen, NJ, USA) using the following sequence for 6 cycles at a 12 CPS rate: pre‐cool for 3 min, run for 1 min 30 s, cool for 1 min. The resulting samples were stored at −80°C until extraction.

### 3.2 UHPLC‐TripleTOF‐ESI‐MSMS analysis

Each sample (30 mg) and 80% aqueous MeOH (1 mL) in a 2‐mL Eppendorf tube was ultrasonically extracted for 30 min at 35°C and were centrifuged (Smart R17, Hanil Science industrial Co., Ltd., Seoul, Korea) at 15,000 rpm under 4°C for 15 min. Then, each supernatant was transferred to another 2‐mL Eppendorf tube and dried in a vacuum centrifuge dryer at 20,000 rpm at 4°C until the sample was dry. Obtained samples were dissolved at 1000 ppm in 80% aqueous MeOH and were filtered (0.22‐μm membrane filter, Woongki Science Co., Ltd., Seoul, Korea).

HPLC‐grade methanol (MeOH), acetonitrile (ACN), and H_2_O were purchased from Merck (Darmstadt, Germany). Formic acid (FA) was obtained from Sigma‐Aldrich (St. Louis, MO, USA). An Ultimate 3000 ultrahigh performance liquid chromatography (UHPLC) system (Thermo Fisher Scientific Inc., Sunnyvale, CA, USA) was used in this study to profile metabolites of *A. distichum* flowers. The system consisted of an Ultimate 3000 autosampler, a column oven, a high‐pressure solvent delivery pump, an automatic degasser, and a diode array detector. Chromatographic separations of metabolites were performed using a Waters Cortecs UPLC T3 column (2.1 mm × 150 mm × 1.6 μm, Waters Co., Milford, MA, USA). The flow rate was set to 0.4 mL/min. A 5‐μL aliquot of 1000 ppm of *A. distichum* flower 80% MeOH extract was injected, and the column oven was set at 45°C. The mobile phase (0.1% FA in H_2_O, solvent A; 0.1% FA in acetonitrile, solvent B) was eluted with the following elution gradient of B: 5% (0.01 min) → 5% (1 min) → 25% (6 min) → 25% (7 min) → 95% (12 min) → 95% (15 min) → 5% (16 min) → 5% (20 min). MS and MS/MS detection were conducted on a Triple TOF™ 5600^+^ (AB SCIEX, Los Angeles, CA, USA) operating in a negative ion electrospray mode. The mass scan type was full scan, and we employed information dependent acquisition (IDA) scanning with a nebulizing gas at 3.44738 bar, a heating gas at 3.44738 bar, and a curtain gas at 1.72369 bar. The desolation temperature was 500°C, the ion spray voltage was 4.5 kV, the collision energy was −35 ± 15 eV, and the collision gas was N_2_. The mass range was set at *m/z* 100–2000 Da for MS scans and MS/MS scans.

Raw chromatographic data acquired from LC/MS analysis were processed by PeakView 2.2 (SCIEX, Framingham, MA, USA) and Scafford Elements version 2.2.1 (Proteome Software, Inc, Portland, OR, USA), in which automatic peak detection and mass spectrum deconvolution were performed with references to the NIST Library (ver. 2017 for Elements), the Human Metabolome Database (HMDB, http://www.hmdb.ca), and the MoNA export LC‐MS, MS‐MS Library.

### 3.3 Gas chromatography–mass spectrometry (GC‐MS) analysis

Each sample (50 mg) and 100% MeOH (1 mL) in a 2‐mL Eppendorf tube was ultrasonically extracted for 30 min at 37°C and centrifuged (Smart R17, Hanil Science Industrial Co., Ltd., Seoul, Korea) at 14,000 rpm under 4°C for 10 min. Then, 100 μL of supernatant was transferred to another 2‐mL Eppendorf tube. The extract was dried in a vacuum centrifuge dryer at 20,000 rpm under 4°C until it was dry. For derivatization, 30 μL of methoxyamine hydrochloride in pyridine (20 mg/mL) was added as the first derivatizing agent. The mixture was incubated in a water bath at 30°C for 90 min. A second derivatizing agent, 50 μL of BSTFA containing 1% TMC, was added and incubated in a dry oven at 70°C for 30 min. Then, 5 μL of fluoranthene (500 ppm in pyridine) was added in each sample as an internal standard (Figure S14.).

All chemicals used in this study were analytical grade. Methanol was used as an extraction solvent, fluoranthene (diluted with pyridine to a concentration of 0.5 mg/mL), *n*‐alkane standards (C_7_–C_30_), and pyridine were purchased from Sigma‐Aldrich Chemical Co. (WO, USA). Methoxyamine hydrochloride and *N*,*O*‐bis(trimethylsilyl) trifluoroacetamide (BSFTA) was purchased from Sigma (WO, USA). A Thermo Scientific Trace™ 1300 (Thermo Fisher Scientific Inc., USA) gas chromatography (GC) instrument was used in this study to identify primary metabolites of *A. distichum* flowers. The primary metabolites were separated using a DB‐5 MS column (60 m × 0.25 mm internal diameter × 0.25 μm film thickness). Helium was used as the carrier gas at a constant flow rate of 1.5 mL/min. The oven temperature condition started at 50°C for 2 min, and then it was programmed to increase from 50°C to 180°C at a rate of 5°C/min, then it was held for 8 min, then heated from 180°C to 210°C at a rate of 2.5°C/min, and then from 210°C to 325°C at a rate of 5°C/min and was finally held for 20 min. A 1‐μL sample was injected with a 20:1 (*v/v*) split ratio. The GC‐MS transfer line temperature was set at 300°C. The triple quadrupole mass spectrometer (TSQ 8000, Thermo Fisher Scientific Inc.) operated in scan mode at 70 eV, and the electron ionization (EI) source was kept at 270°C. Two scans per second were recorded over the mass range *m/z* of 35–650 Da. The identification of metabolites was confirmed by comparing their spectra and retention indices (RI) with standards from the NIST 2.0 library. Quantitative analysis by the peak area normalization method was conducted to determine the relative amounts using an internal standard. RI were calculated using the following equation (Kováts, 1958): RI = 100 × n + [100 × (*t*_x_ − *t*_n_)]/(*t*_n + 1_ − *t*_n_). Here, x is the targeted compound, n is the number of carbon atoms of the *n*‐alkane eluted before x. n + 1 is the number of carbon atoms of the *n*‐alkane eluted after x, *t*_x_ is the retention time of x, *t*_n_ is the retention time of n, and *t*_n + 1_ is the retention time of n + 1.

Raw chromatographic data acquired from triple quadrupole GC/MS analysis were processed using Xcalibur 3.1 software (Thermo Finnigan Corporation, San Jose, CA, USA), in which automatic peak detection and mass spectrum deconvolution (compound identification) were performed with references to library NIST 2.0.

### 3.4 NMR analysis

Fifty milligrams of each ground sample and 1.5 mL of a 1:1 mixture of deuterated methanol (MeOD) and deuterated water (D_2_O) in a 2 mL Eppendorf tube were vortexed for 1 min. After vortexing, each tube was ultrasonically extracted for 20 min at 30°C and centrifuged (Smart R17, Hanil Science Industrial Co., Ltd., Seoul, Korea) at 15,000 rpm and 4°C for 15 min. Then, 750 μL of each supernatant was transferred into Norell® Standard Series™ 5 mm NMR tubes (for 600 MHz, Norell, Inc. Mays Landing, NJ, USA) for NMR measurements (Figure S15.).

D_2_O [99.9% atom% D, contains 0.05% (*v/v*) 3‐(trimethylsilyl)‐propionic‐2,2,3,3‐*d*_4_ acid sodium salt (TSP) as an internal standard], methanol‐*d*_4_ (99.8 atom% D), and other deuterated NMR solvents (99.8 atom% D) were purchased from Sigma Aldrich Co. Ltd (St. Louis, MO, USA). 1D (^1^H‐ and ^13^C‐) and 2D (COSY, HSQC, HMBC, TOCSY, and *J*‐reserved) NMR spectra of *A. distichum* flowers were recorded on a Bruker Advance 600 spectrometer (Billerica, MA, USA) operating at 600.13 MHz with a 5‐mm TXI probe at 298 K. A presaturation pulse sequence of all 1D NMR spectra with zg30 without residual H_2_O suppression via low power selective irradiation of the H_2_O frequency during mixing and recycle delay were acquired. Measurements were made using a 20.03 ppm (12019.23 Hz) spectral width, a 5.0‐s repetition time, and a pulse width of 90.74° (flip angle). Thirty‐two transients were collected with 63,536 data points at an acquisition time of 2.73 s. Two dummy scans were made prior to the 128 recorded scans. The free induction decays (FIDs) were Fourier transformed with a line‐broadening function of 0.37 Hz. The baselines of ^1^H‐ and ^13^C‐NMR spectra were manually referenced to the internal standard (TSP, *δ*_H_ and *δ*_C_ 0.00 ppm). The COSY spectra with a pulse sequence of cosydfgpph19 were acquired with a covering spectral width of 13.02 ppm (7812.50 Hz) in both dimensions. The data matrix had 2048 × 128 points, there were 32 transients per increment at an acquisition time of 0.13 s. Eight dummy scans were obtained prior to 64 recorded scans. The sine bell‐shaped window function (SSB = 2.0) was applied for COSY spectra processing. The TOCSY spectra with a pulse sequence of mlevphpp were acquired with a spanning spectral width of 8.62 ppm (5175.98 Hz) in both dimensions. The data matrix had 1024 × 1024 points, there were 203 transients per increment at an acquisition time of 0.20 s. Sixteen dummy scans were conducted prior to 48 recorded scans. The sine bell‐shaped window function (SSB = 2.0) was applied for TOCSY spectra processing. HSQC spectra, in which the pulse sequence was hsqcedetgpsp.3, were obtained with a spectral width of 180.00 ppm (27,164.95 Hz) in F1 and 15.02 ppm (9014.42 ppm) in F2. The data matrix contained 2048 × 256 points with 203 transients per increment. The sine bell‐shaped window function (SSB of F1 = 2.0, SSB of F2 = 0.0) was applied for F1 and F2 dimensions of HSQC spectra processing. HMBC spectra with a pulse sequence of hmbcetgpl3nd were acquired with a spanning spectral width of 13.02 ppm (7812.50 Hz) in both dimensions of 230.00 ppm (34711.47 Hz) in F1 and 15.02 ppm (9014.42 ppm) in F2. The data matrix had 4096 × 512 points with 203 transients per increment. The sine bell‐shaped window function (SSB of F1 = 2.0, SSB of F2 = 0.0) was applied for F1 and F2 dimensions of HMBC spectra processing. 2D‐dimensional *J*‐reserved spectra with a pulse sequence of jresgpprqf were acquired with a spectral width of 0.13 ppm (77.98 Hz) in F1 and 16.62 ppm (9973.40 ppm) in F2. The data matrix had 8192 × 128 points with 128 transients per increment. A sine bell‐shaped window function (SSB = 0.0) was applied for *J*‐reserved spectra processing. 2D‐dimensional *J*‐resolved spectra were tilted along the rows by 45° relative to the frequency axis and were made symmetrical about F1.

Acquisition and processing of 1D and 2D NMR spectra of *A. distichum* flowers were carried out with AMIX ver. 3.6.8 software (Bruker BioSpin, Germany), Topspin ver. 4.06 (Bruker), and MestReNova ver. 5.3.1–4696 (Mestrelab Research S.L., Santiago de Compostela, Spain). Spectral intensities of ^1^H‐NMR spectra corresponding to the region of *δ*_H_ 10.00–0.50 were divided into equal widths (0.04 ppm). Due to the residual signal of deuterated water, *δ*_H_ values of 4.86–4.66 ppm (region of water) was excluded in these experiments. Metabolite profiling was performed using Chenomx NMR suite ver. 15.0 (Chenomx, Edmonton, Canada) and AssureNMR ver 2.1 (Bruker) software and was confirmed with 2D NMR spectra and literature.

### 3.5 Statistical and multivariate analysis

PCA and PLS‐DA were then chosen to create a prediction model. SIMCA version 14.1 (Umetrics, Umeå, Sweden) and MetaboAnalyst 4.0 (http://www.metaboanalyst.ca) were initially employed to understand the relationship expressed in terms of similarity or dissimilarity among groups of multivariate data. Statistical analysis was performed using GraphPad Prism software version 7.00 (GraphPad Software, Inc., San Diego, CA, USA). Significance was estimated using repeated one‐way ANOVA followed by Tukey's test. Data were presented as mean ± standard error.

## 4 CONCLUSION

**FIGURE 9:**
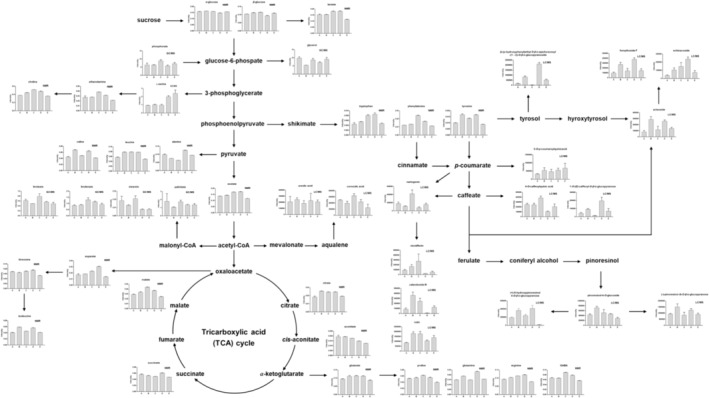
Schematic diagram of the metabolic pathway and relative levels on five variants of *Abeliophyllum distichum* flowers. (a) White miseon, (b) pink miseon, (c) ivory miseon, (d) blue miseon, (e) round miseon.

In this study, we used three representative metabolomics flatforms (GC/MS, LC/MS, and NMR) to understand the chemical composition of primary metabolites and secondary metabolites in five variants of *A. distichum* flowers. We revealed their metabolic flux and reconstructed the metabolic pathways responsible for dissimilarities in the morphological characteristics of five variants of *A. distichum* flowers. As shown in Figure 9, the metabolic pathways of *A. distichum* flowers were established using variables with VIP values greater than 0.7 in each metabolomic platform (i.e., LC/MS, GC/MS, and NMR). Schemes of metabolic pathways were revealed according to the Kyoto Encyclopedia of Genes and Genomes (KEGG) (http://www.genome.jp/kegg). According to the shikimate pathway (Figure 9), tyrosine plays a significant role in the biosynthesis of phenolic glycosides, phenylpropanoid glycosides, and lignans. Among them, phenolic glycosides (1‐*O*‐(*E*)‐phenylethyl‐*β*‐D‐glucofuranosyl‐*O*‐(1 → 2)‐*β*‐D‐glucopyranoside and 1‐*O*‐(*E*)‐caffeoyl‐*β*‐D‐glucopyranose) is contained in a large quantity in blue miseon. This indicates that phenolic glycosides are related to the color of the sepals. Phenylpropanoid glycosides and lignans were deeply related to the morphological characteristics of pink and blue miseon. Variants having colored petals showed high concentrations of amino acids (tryptophan, phenylalanine, and tyrosine) derived from the shikimate pathway. On the other side, alanine derived from pyruvate (the next step in the shikimate pathway) showed low concentrations in colored variants. Also, phenylalanine derived from shikimic acid was related to the biosynthesis of flavonoids. Naringenin, a precursor of flavonoids, was significant in ivory miseon. This flavanone was associated with yellowish pigments in these flowers. Also, flavonol glycosides, which are the next step in the flavonoid biosynthesis of naringenin, were highly involved in colored variants (ivory and pink miseon). These components were deeply related to the colors of the petals. Serine concentration was relatively higher in round miseon than in other variants. Therefore, round miseon contains relatively low contents of both acetate and organic acids and amino acids derived from the tricarboxylic acid (TCA) cycle. Therefore, we proposed that the content of serine and organic acids and amino acids derived from TCA cycle were related to fruit shape. The extensive integration of *A. distichum* metabolic networks revealed by metabolomics flatforms allowed us better to understand the chemical compositions and dissimilarities in morphological characteristics.

## AUTHOR CONTRIBUTIONS

Yeong‐Geun Lee, Nam‐In Baek, and Se Chan Kang designed the research; Yeong‐Geun Lee, Jeong Eun Kwon, and Won‐Sil Choi performed the experiments; Yeong‐Geun Lee, Jeong Eun Kwon, Won‐Sil Choi, and Nam‐In Baek analyzed data; and Yeong‐Geun Lee wrote the paper. All authors contributed to the article and approved the submitted version.

## CONFLICT OF INTEREST STATEMENT

The authors declare that the research was conducted in the absence of any commercial or financial relationships that could be construed as a potential conflict of interest.

## Supporting information


**Figure S1.** ^1^H‐NMR spectra of flowers of white miseon (*Abeliophyllum distichum*) analyzed with various deuterium NMR solvents.
**Figure S2.** Representative ^1^H‐^1^H COSY spectrum of flowers of white miseon (*Abeliophyllum distichum*) analyzed with 1:1 mixture of CD_3_OD and D_2_O.
**Figure S3.** Representative HSQC spectrum of flowers of white miseon (*Abeliophyllum distichum*) analyzed with 1:1 mixture of CD_3_OD and D_2_O.
**Figure S4.** Representative HMBC spectrum of flowers of white miseon (*Abeliophyllum distichum*) analyzed with 1:1 mixture of CD_3_OD and D_2_O.
**Figure S5.** Representative TOCSY spectrum of flowers of white miseon (*Abeliophyllum distichum*) analyzed with 1:1 mixture of CD_3_OD and D_2_O.
**Figure S6.** Representative *J*‐reserved spectrum of flowers of white miseon (*Abeliophyllum distichum*) analyzed with 1:1 mixture of CD_3_OD and D_2_O.
**Figure S7.** PCA loading plot obtained from LC–MS data on five variants of *Abeliophyllum distichum* flowers.
**Figure S8.** PCA biplot obtained from LC–MS data on five variants of *Abeliophyllum distichum* flowers. (A) White miseon, (B) pink miseon, (C) ivory miseon, (D) blue miseon, (E) round miseon.
**Figure S9.** Dendrogram of hierarchical cluster analysis of the PLS‐DA result obtained from LC–MS data on five variants of *Abeliophyllum distichum* flowers. (A) White miseon, (B) pink miseon, (C) ivory miseon, (D) blue miseon, (E) round miseon.
**Figure S10.** PCA loading plot obtained from GC/MS results on five variants of *Abeliophyllum distichum* flowers.
**Figure S11.** PLS‐DA score plot obtained from GC/MS results on five variants of *Abeliophyllum distichum* flowers. (A) White miseon, (B) pink miseon, (C) ivory miseon, (D) blue miseon, (E) round miseon.
**Figure S12.** PLS‐DA loading plot obtained from GC/MS results on five variants of *Abeliophyllum distichum* flowers.
**Figure S13.** PCA loading plots obtained from NMR result on five variants of *Abeliophyllum distichum* flowers.
**Figure S14.** Simplified experimental procedures of GC/MS metabolomics of *Abeliophyllum distichum* flowers.
**Figure S15.** Simplified experimental procedures of NMR metabolomics of *Abeliophyllum distichum* flowers.
