# Supplementary material for: Deciphering chemical diversity among five variants of Abeliophyllum distichum flowers through metabolomics analysis
Source: Plant Direct. 2024 Sep 19;8(9):e616. doi: 10.1002/pld3.616 (PMC11411454; doi:10.1002/pld3.616)
Supplement: Supplementary file 1 — Figure S1. 1H‐NMR spectra of flowers of white miseon (Abeliophyllum distichum) analyzed with various deuterium NMR solvents. Figure S2. Representative 1H‐1H COSY spectrum of flowers of white miseon (Abeliophyllum distichum) analyzed with 1:1 mixture of CD3OD and D2O. Figure S3. Representative HSQC spectrum of flowers of white miseon (Abeliophyllum distichum) analyzed with 1:1 mixture of CD3OD and D2O. Figure S4. Representative HMBC spectrum of flowers of white miseon (Abeliophyllum distichum) analyzed with 1:1 mixture of CD3OD and D2O. Figure S5. Representative TOCSY spectrum of flowers of white miseon (Abeliophyllum distichum) analyzed with 1:1 mixture of CD3OD and D2O. Figure S6. Representative J‐reserved spectrum of flowers of white miseon (Abeliophyllum distichum) analyzed with 1:1 mixture of CD3OD and D2O. Figure S7. PCA loading plot obtained from LC–MS data on five variants of Abeliophyllum distichum flowers. Figure S8. PCA biplot obtained from LC–MS data on five variants of Abeliophyllum distichum flowers. (A) White miseon, (B) pink miseon, (C) ivory miseon, (D) blue miseon, (E) round miseon. Figure S9. Dendrogram of hierarchical cluster analysis of the PLS‐DA result obtained from LC–MS data on five variants of Abeliophyllum distichum flowers. (A) White miseon, (B) pink miseon, (C) ivory miseon, (D) blue miseon, (E) round miseon. Figure S10. PCA loading plot obtained from GC/MS results on five variants of Abeliophyllum distichum flowers. Figure S11. PLS‐DA score plot obtained from GC/MS results on five variants of Abeliophyllum distichum flowers. (A) White miseon, (B) pink miseon, (C) ivory miseon, (D) blue miseon, (E) round miseon. Figure S12. PLS‐DA loading plot obtained from GC/MS results on five variants of Abeliophyllum distichum flowers. Figure S13. PCA loading plots obtained from NMR result on five variants of Abeliophyllum distichum flowers. Figure S14. Simplified experimental procedures of GC/MS metabolomics of Abeliophyllum distichum flowers. Figure [file PLD3-8-e616-s001.docx]

The Plant Journal Supporting Information

Deciphering Chemical Diversity among Five Variants of *Abeliophyllum distichum* Flowers through Metabolomics Analysis

Yeong-Geun Lee ^1^, Jeong Eun Kwon ^1^, Won-Sil Choi ^2^, Nam-In Baek ^1^, Se Chan Kang ^1,*^

^1^Graduate School of Biotechnology and Department of Oriental Medicine Biotechnology, Kyung Hee University, Yongin 17104, Korea

^2^National Instrumentation Center for Environmental Management, Seoul National University, Seoul 08826, Korea

* Correspondence: Se Chan Kang (e-mail: sckang@khu.ac.kr)

**
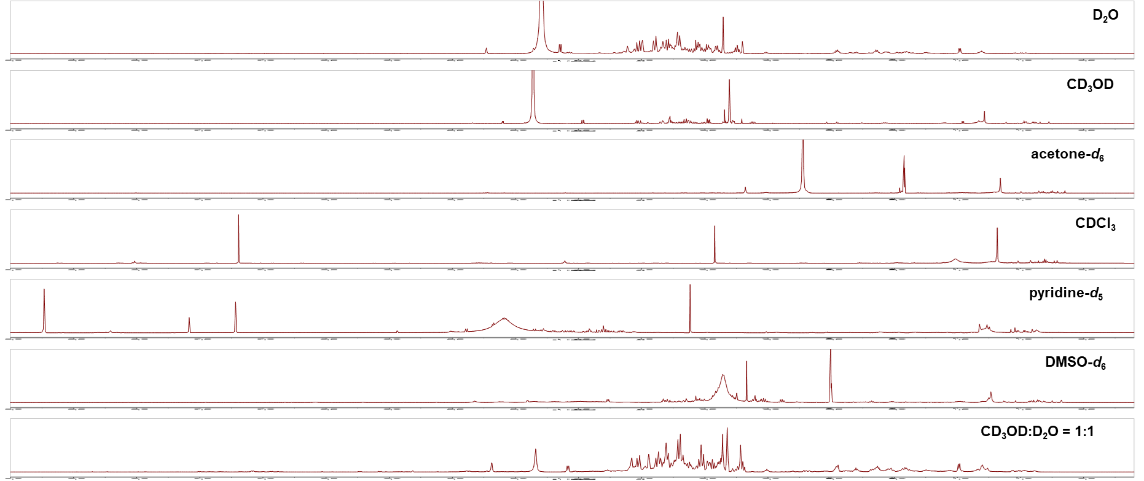
**

**Figure S1. ^1^H-NMR spectra of flowers of white miseon (*Abeliophyllum distichum*) analyzed with various deuterium NMR solvents.**

**
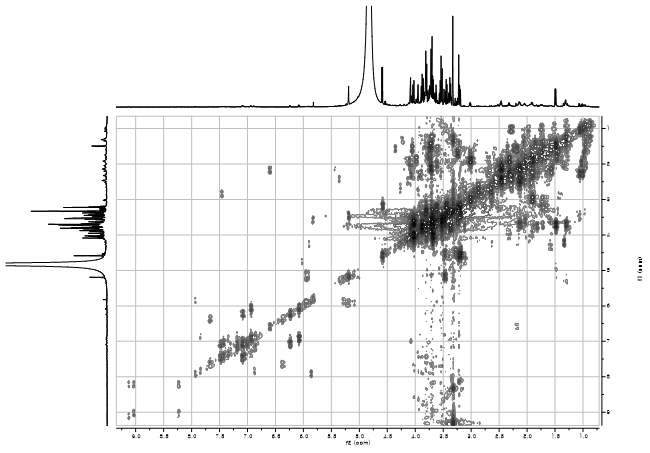
**

**Figure S2. Representative ^1^H-^1^H COSY spectrum of flowers of white miseon (*Abeliophyllum distichum*) analyzed with 1:1 mixture of CD_3_OD and D_2_O.**

**Figure S3. Representative HSQC spectrum of flowers of white miseon (*Abeliophyllum distichum*) analyzed with 1:1 mixture of CD_3_OD and D_2_O.**

**Figure S4. Representative HMBC spectrum of flowers of white miseon (*Abeliophyllum distichum*) analyzed with 1:1 mixture of CD_3_OD and D_2_O.**

**Figure S5. Representative TOCSY spectrum of flowers of white miseon (*Abeliophyllum distichum*) analyzed with 1:1 mixture of CD_3_OD and D_2_O.**

**Figure S6. Representative *J*-reserved spectrum of flowers of white miseon (*Abeliophyllum distichum*) analyzed with 1:1 mixture of CD_3_OD and D_2_O.**


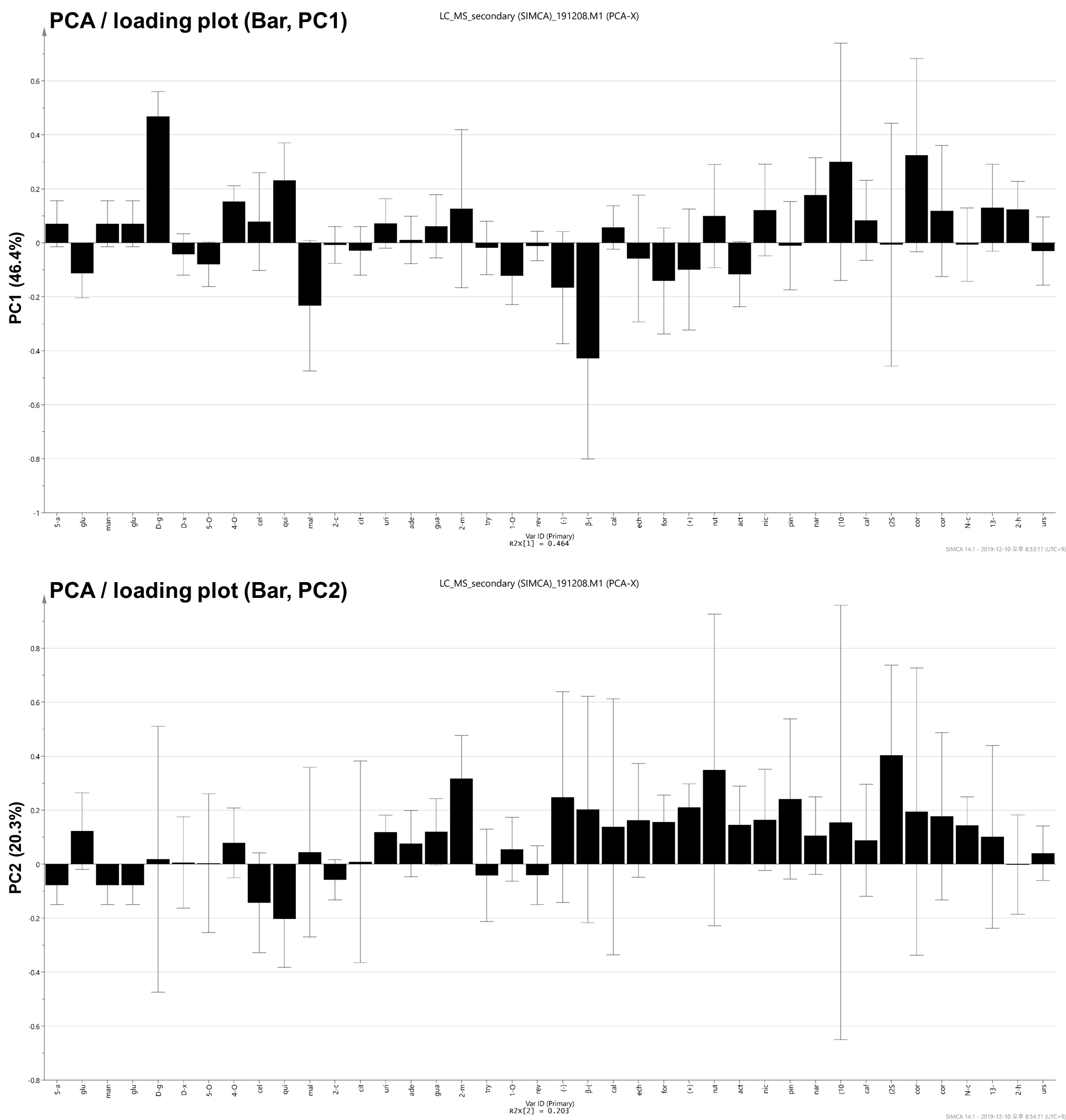


**Figure S7. PCA loading plot obtained from LC-MS data on five variants of *Abeliophyllum distichum* flowers.**


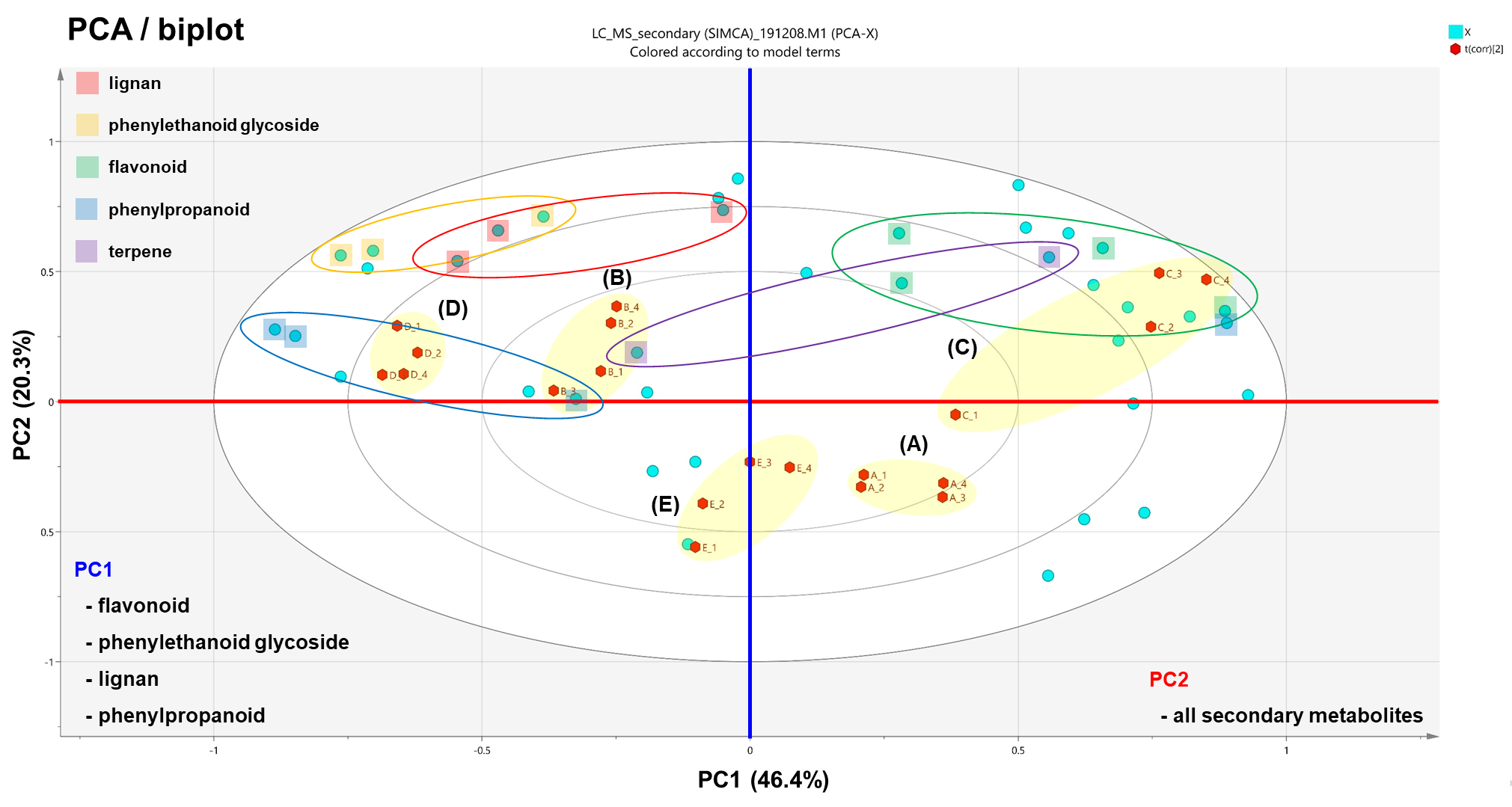


**Figure S8. PCA biplot obtained from LC-MS data on five variants of *Abeliophyllum distichum* flowers.** (A) White miseon, (B) pink miseon, (C) ivory miseon, (D) blue miseon, (E) round miseon.


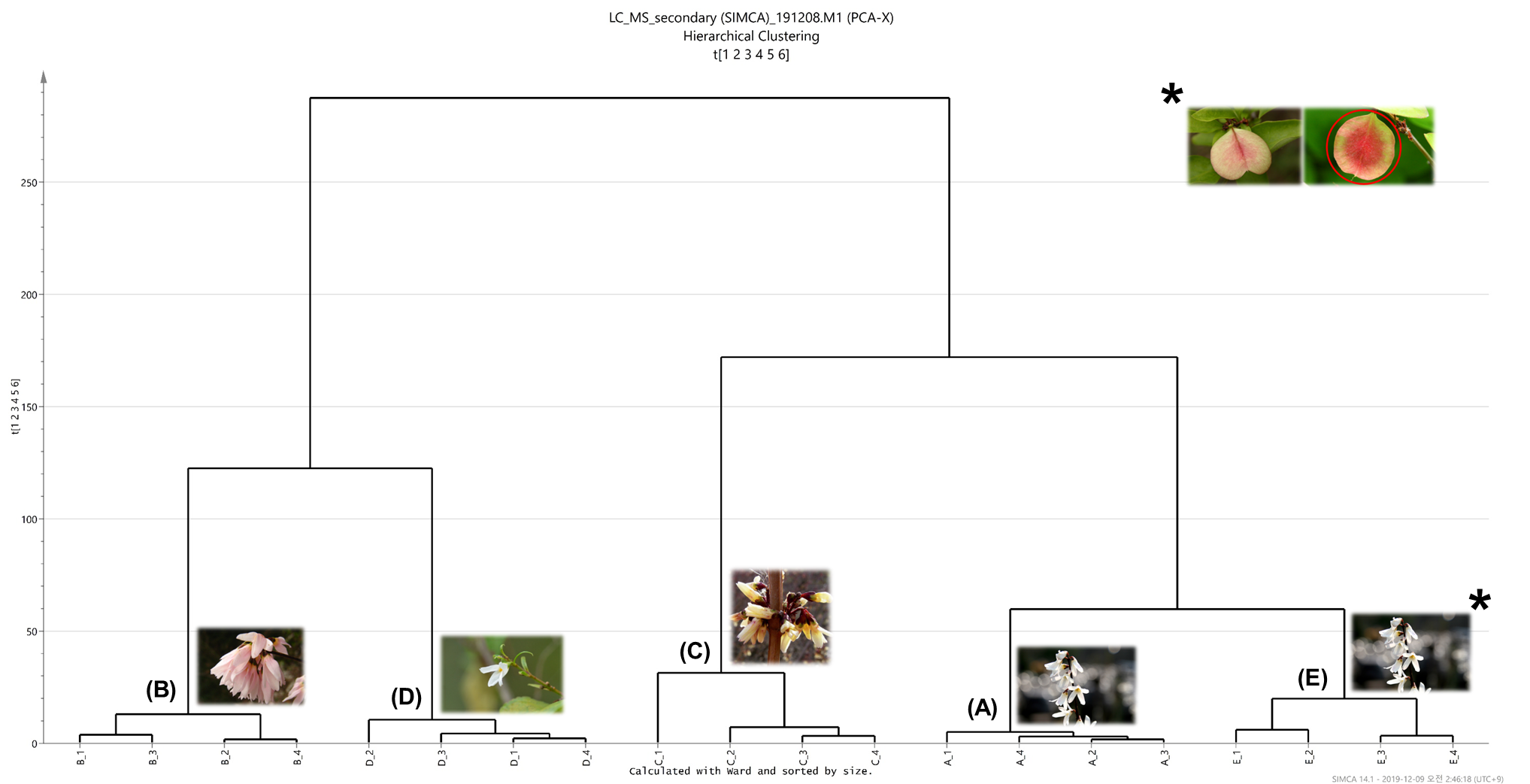


**Figure S9. Dendrogram of hierarchical cluster analysis of the PLS-DA result obtained from LC-MS data on five variants of *Abeliophyllum distichum* flowers.** (A) White miseon, (B) pink miseon, (C) ivory miseon, (D) blue miseon, (E) round miseon.


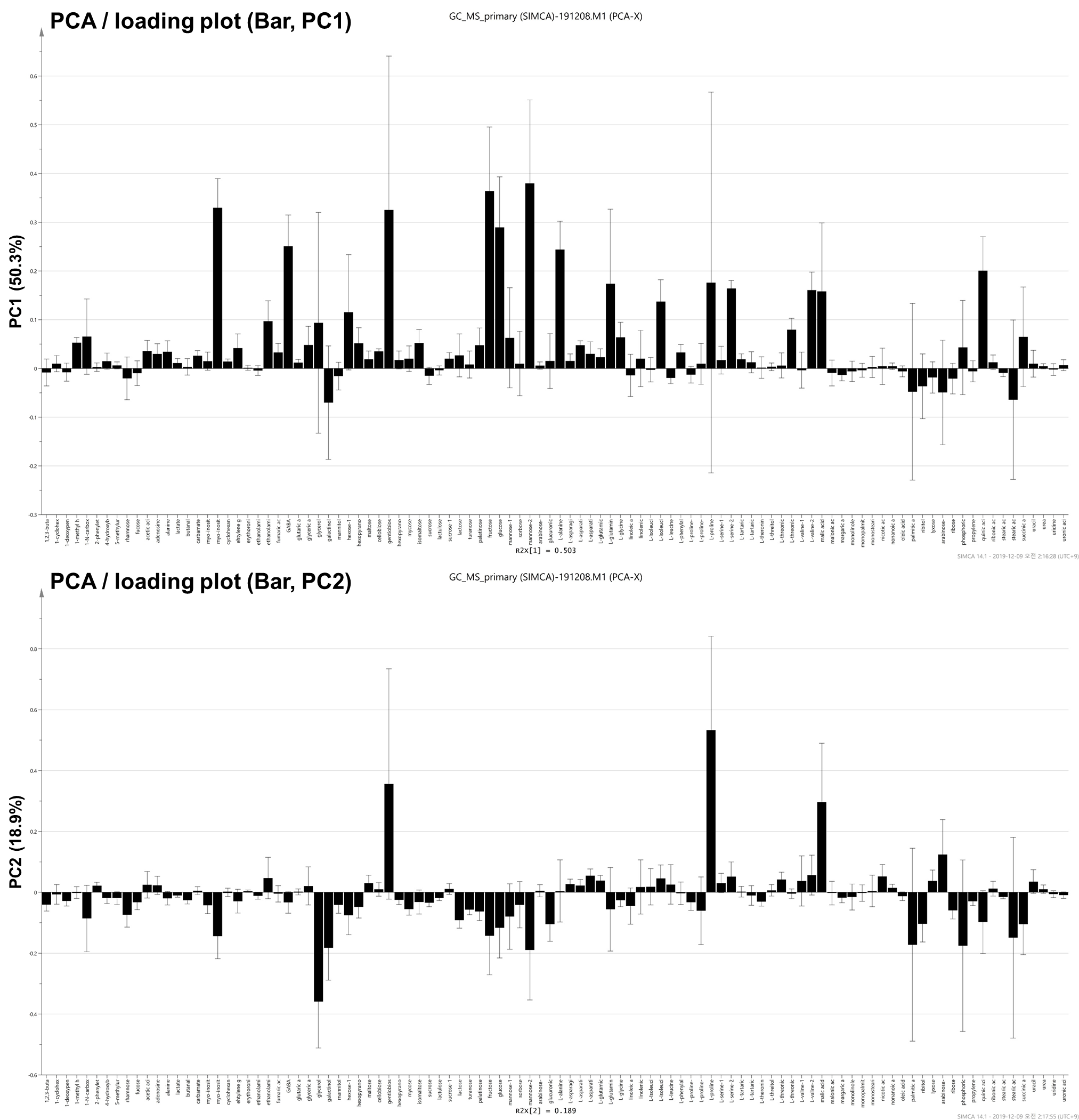


**Figure S10. PCA loading plot obtained from GC/MS results on five variants of *Abeliophyllum distichum* flowers.**


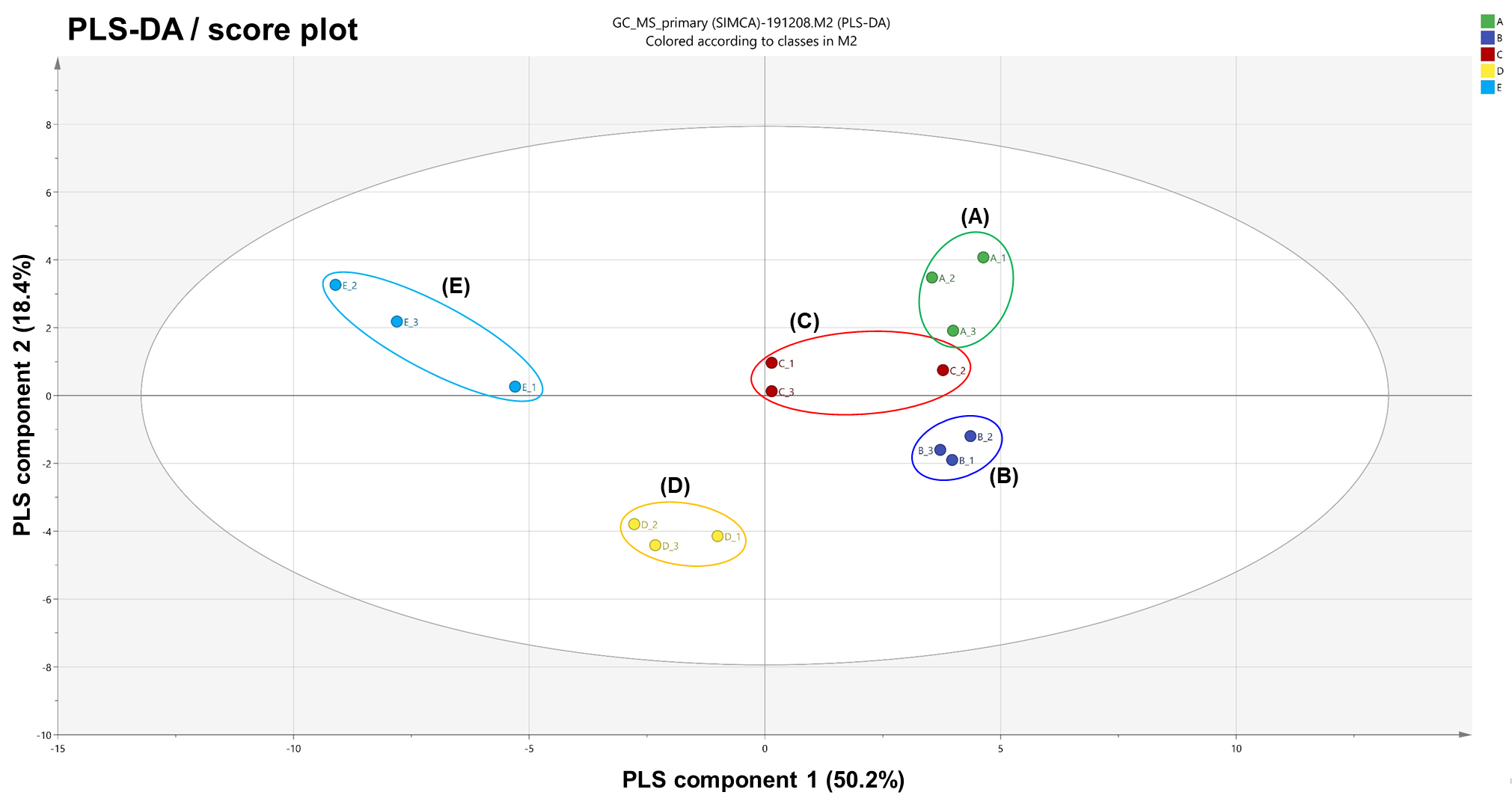


**Figure S11. PLS-DA score plot obtained from GC/MS results on five variants of *Abeliophyllum distichum* flowers.** (A) White miseon, (B) pink miseon, (C) ivory miseon, (D) blue miseon, (E) round miseon.


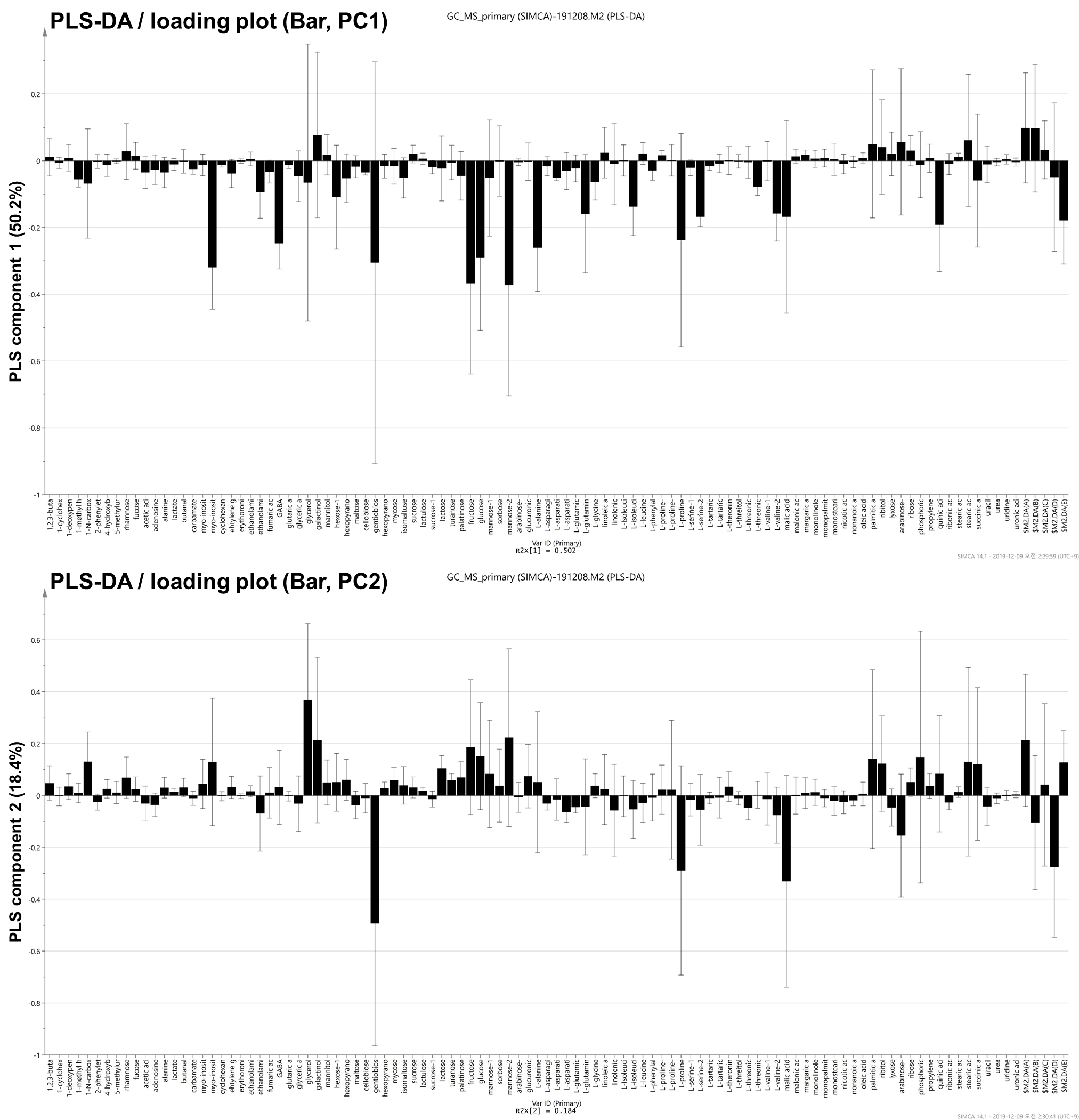


**Figure S12. PLS-DA loading plot obtained from GC/MS results on five variants of *Abeliophyllum distichum* flowers.**


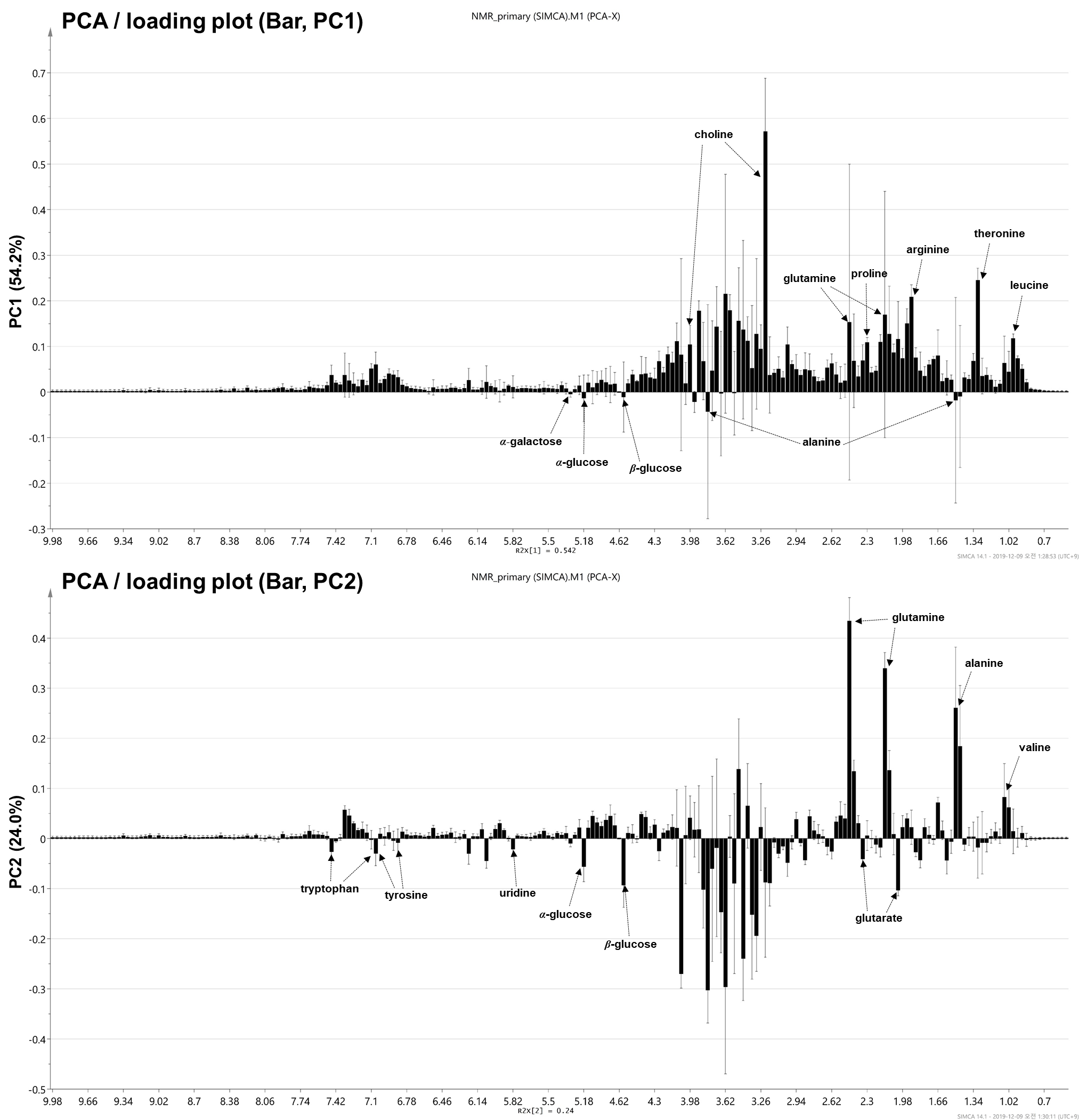


**Figure S13. PCA loading plots obtained from NMR result on five variants of *Abeliophyllum distichum* flowers**.

**
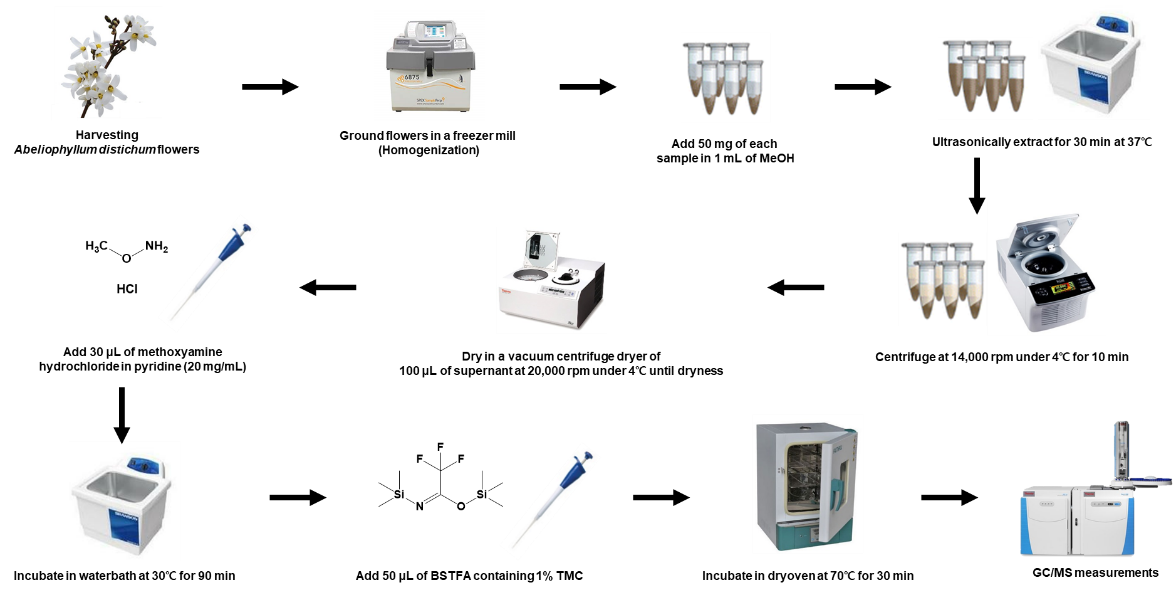
**

**Figure S14. Simplified experimental procedures of GC/MS metabolomics of *Abeliophyllum distichum* flowers.**


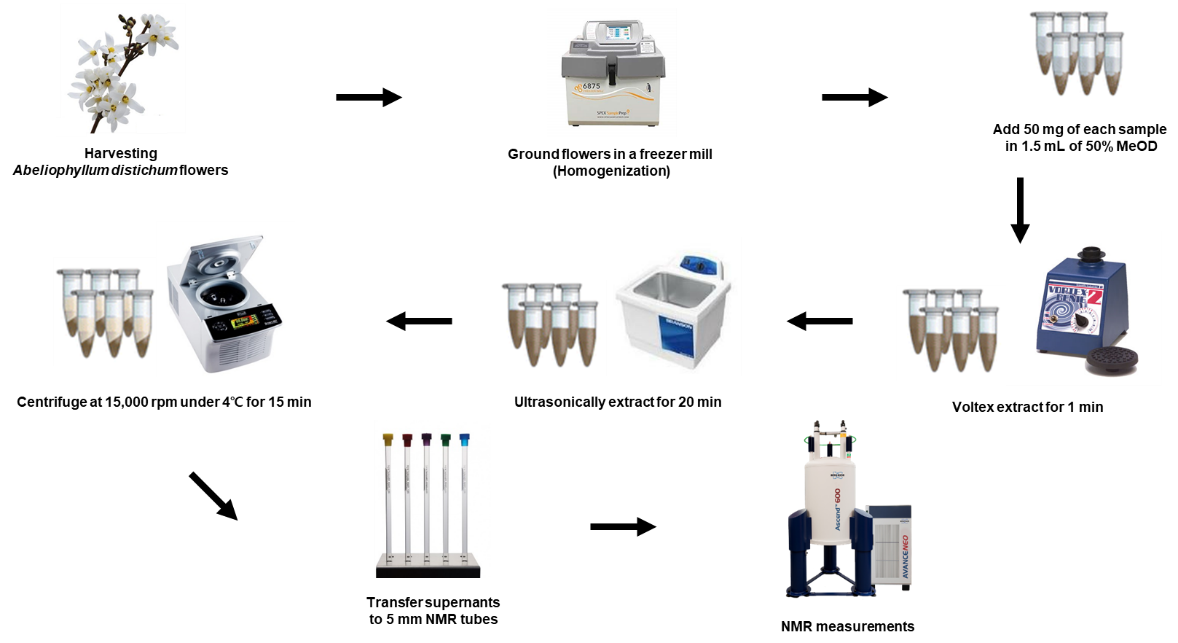


**Figure S15. Simplified experimental procedures of NMR metabolomics of *Abeliophyllum distichum* flowers.**
